# The regulation of cardiac intermediary metabolism by NADPH oxidases

**DOI:** 10.1093/cvr/cvac030

**Published:** 2022-03-24

**Authors:** Adam A Nabeebaccus, Christina M Reumiller, Jie Shen, Anna Zoccarato, Celio X C Santos, Ajay M Shah

**Affiliations:** School of Cardiovascular Medicine and Sciences, King’s College London British Heart Foundation Centre of Excellence, James Black Centre, 125 Coldharbour Lane, London SE5 9NU, UK; School of Cardiovascular Medicine and Sciences, King’s College London British Heart Foundation Centre of Excellence, James Black Centre, 125 Coldharbour Lane, London SE5 9NU, UK; School of Cardiovascular Medicine and Sciences, King’s College London British Heart Foundation Centre of Excellence, James Black Centre, 125 Coldharbour Lane, London SE5 9NU, UK; School of Cardiovascular Medicine and Sciences, King’s College London British Heart Foundation Centre of Excellence, James Black Centre, 125 Coldharbour Lane, London SE5 9NU, UK; School of Cardiovascular Medicine and Sciences, King’s College London British Heart Foundation Centre of Excellence, James Black Centre, 125 Coldharbour Lane, London SE5 9NU, UK; School of Cardiovascular Medicine and Sciences, King’s College London British Heart Foundation Centre of Excellence, James Black Centre, 125 Coldharbour Lane, London SE5 9NU, UK

**Keywords:** NADPH oxidases, Cardiac metabolism, Intermediary metabolism, Redox signalling

## Abstract

NADPH oxidases (NOXs), enzymes whose primary function is to generate reactive oxygen species, are important regulators of the heart’s physiological function and response to pathological insults. The role of NOX-driven redox signalling in pathophysiological myocardial remodelling, including processes such as interstitial fibrosis, contractile dysfunction, cellular hypertrophy, and cell survival, is well recognized. While the NOX2 isoform promotes many detrimental effects, the NOX4 isoform has attracted considerable attention as a driver of adaptive stress responses both during pathology and under physiological states such as exercise. Recent studies have begun to define some of the NOX4-modulated mechanisms that may underlie these adaptive responses. In particular, novel functions of NOX4 in driving cellular metabolic changes have emerged. Alterations in cellular metabolism are a recognized hallmark of the heart’s response to physiological and pathological stresses. In this review, we highlight the emerging roles of NOX enzymes as important modulators of cellular intermediary metabolism in the heart, linking stress responses not only to myocardial energetics but also other functions. The novel interplay of NOX-modulated redox signalling pathways and intermediary metabolism in the heart is unravelling a new aspect of the fascinating biology of these enzymes which will inform a better understanding of how they drive adaptive responses. We also discuss the implications of these new findings for therapeutic approaches that target metabolism in cardiac disease.

## 1. Introduction

The heart must contend with two interconnected functions: (i) to provide continuous, yet rapidly responsive contractile activity to pump blood to the tissues to meet varying demand and (ii) to undergo chronic remodelling of structure and function to maintain homeostasis and adapt to longer-term physiological stressors. For instance, during acute physiological fluctuations (e.g. fight or flight responses), high contractile activity and energetic efficiency are needed, whereas more chronic physiological stressors such as pregnancy, exercise, and growth from childhood to adulthood also require adaptive myocardial hypertrophy.^1^ To fulfil both functions, the heart has evolved elaborate metabolic adaptations to alter its substrate utilization, in the form mainly of carbohydrates and fatty acids, both to deliver high aerobic energy production for contractile function and to maintain cellular structures and integrity and any requirements for remodelling.^2,3^ Alterations to intermediary metabolism, which describes how substrates and their downstream metabolite intermediates integrate in various metabolic pathways to maintain physiological function, may be key to understanding pathological states such as hypertensive heart disease, ischaemia, and diabetes or obesity-related cardiomyopathies that lead to heart failure.^2,4^

The failing heart is characterized by several changes in metabolism. These include a reduction in glycolysis and altered glucose oxidation and fatty acid oxidation leading to the energetic deficit and pump failure,^5^ as well as interrelated changes affecting key cellular functions such as excitation–contraction coupling, cell hypertrophy, cell viability, fibrosis, and other processes.^2,4^ Emerging data indicate that enzymes of the NADPH oxidase (NOX) family are key regulators of intermediary metabolic processes in the heart. Here, we review the main NOX isoforms that mediate the redox regulation of pathways involved in cardiac intermediary metabolism. We highlight the emerging links to heart function including effects on the cellular integrated stress response (ISR), mitochondrial function, calcium homeostasis, cellular differentiation, and hypertrophy. A better understanding of this complex regulation by NOX enzymes will provide new mechanistic insights into cardiac metabolic reprogramming and adaption to pathological stress and may aid the development of novel therapeutic strategies for heart disease.

## 2. NOX isoforms and the heart

**Figure 1:**
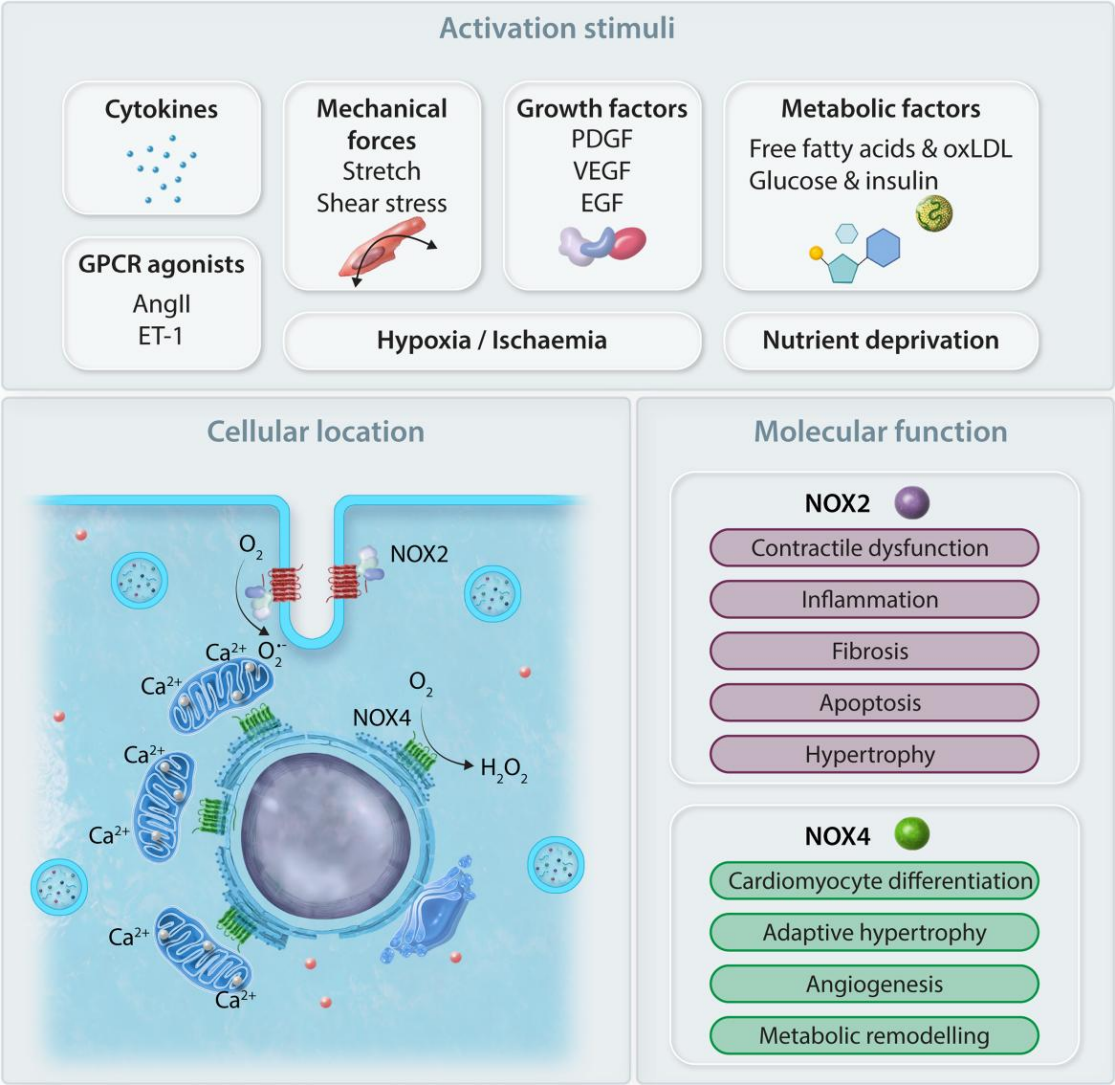
NOX2 and NOX4 are the major isoforms present in the cardiomyocyte. They are activated by a variety of stimuli (e.g. cytokines, mechanical stretch, growth factors, nutrient deprivation, metabolites) and G-protein coupled receptor agonists such as angiotensin II (Ang II) and endothelin-1 (ET-1). Their intracellular locations are also distinct, with NOX2 being located at the sarcolemma and NOX4 located in intracellular membrane compartments (ER/mitochondria/MAM). Another distinction between isoforms is the ROS produced. NOX2 generates superoxide (O_2_^.-^) but NOX4 generates predominantly hydrogen peroxide (H_2_O_2_). It is likely that the differing intracellular locations and ROS-generating properties underlie their functional differences. PDGF, platelet-derived growth factor; VEGF, vascular endothelial growth factor; EGF, epidermal growth factor; oxLDL, oxidized LDL.

NOXs use NADPH to reduce molecular oxygen (O_2_) and form the reactive oxygen species (ROS), superoxide anions (O_2_^.-^), and hydrogen peroxide (H_2_O_2_). The prototype of this family of enzymes is the NOX2 isoform (originally known as gp91phox oxidase). In the last 20 years, seven distinct isoforms (NOX1-5 and DUOX1-2) have been identified in diverse cells and tissues.^6^ The NOX1-4 isoforms associate with p22phox protein to form a membrane-bound heterodimer critical to their catalytic activity while NOX5 and the DUOXs retain the catalytic core but without the requirement for the p22phox subunit. The membrane-spanning NOX-p22phox heterodimer allows for the electron transfer from NADPH to bound FAD, two haem moieties, and then to molecular O_2_ on the other side of the membrane to generate superoxide or hydrogen peroxide. Whereas the activation of NOX1 and NOX2 requires association of the membrane-bound heterodimer with regulatory subunits (*described below for NOX2*), NOX3, and NOX4 have no such requirement. NOX5 is different from the other NOXs in being calcium-activated via its EF-hand motifs at the N-terminus and not requiring binding to p22phox. The reader is directed to several comprehensive reviews describing in detail the structure and biology of the different NOX isoforms.^7,8^ The effects of NOXs in cardiac hypertrophy and failure have also been reviewed.^9,10^ Here, we provide a brief overview of NOX2 and NOX4, which are the main isoforms found in the heart (*Figure 1*), and then focus on their roles in the regulation of intermediary metabolism.

NOX2 is normally quiescent but is activated by stimuli such as G-protein coupled receptor agonists (e.g. angiotensin II, endothelin-1), growth factors, cytokines, and mechanical forces in a process that involves the binding of four regulatory subunits (p67phox, p47phox, p40phox, and Rac1) to the NOX2–p22phox complex at the sarcolemma (including T-tubules) of the cardiomyocyte.^11^ NOX4 is reportedly found in many intracellular locations, notably the endoplasmic reticulum (ER),^12–14^ nucleus,^15^ and mitochondria.^16^ In cardiomyocytes, NOX4 is mainly localized to the ER^13,17^ and possibly mitochondria.^18^ More recently, we have identified NOX4 to be present at ER-mitochondrial contacts known as the mitochondrial-associated membrane (MAM),^14^ which may have important implications for its functional roles in regulating metabolism as well as accounting for prior reports that it is located in mitochondria.

In contrast to NOX2, NOX4 does not require additional regulatory subunits and is constitutively active. Its activity depends upon its abundance which is increased by a wide variety of agonists (such as angiotensin II, platelet-derived growth factor, tumour necrosis factor alpha, and mechanical forces)^19^ and cellular stresses (hypoxia, ER and metabolic stress, serum, and glucose withdrawal) both through transcriptional and post-translational mechanisms.^13^ For instance, NOX4 is a transcriptional target of hypoxia-inducible factor 1-alpha (HIF1α) during hypoxia^20^ and of activating transcription factor 4 (ATF4) during diverse stress situations (*discussed later*). E2F transcription factors also regulate NOX4 expression, at least in vascular smooth muscle cells.^21^ There is also a suggestion that epigenetic mechanisms (enhanced histone acetylation) may increase NOX4 expression.^22^ Post-translational regulation of NOX4 may occur at the level of its protein stability. For example, the binding of the protein Poldip2 to the NOX4-p22phox heterodimer is reported to increase its stability and overall ROS-generating activity.^23^ How the intracellular localization of NOX4 is achieved still requires more investigation. However, interaction with cellular compartment-specific proteins may be at least part of the mechanism. For example, calnexin has been shown to be a specific NOX4-interacting protein and this may explain its localization to the ER^24^ and the MAM.^25^ As will be explored in later sections, such specific targeting of NOX4 may be key to determining its role in metabolism.

Another important difference between NOX4 and NOX2 is that NOX4 produces predominantly H_2_O_2_ (a property that appears to have a specific structural basis), whereas NOX2 generates which can then be dismutated to H_2_O_2_.^26,27^ An especially important implication of this difference is in relation to crosstalk with vascular nitric oxide synthase (NOS) signalling: superoxide reacts rapidly with vasodilator nitric oxide (NO) and inactivates it, whereas H_2_O_2_ can increase endothelial NOS expression and/or have direct vasodilator actions (*further highlighted in the section on endothelial cells later*). NOX4 produces localized low levels (nanomolar range) of H_2_O_2_^14,17,28^ that are implicated in the regulation of redox-sensitive cellular processes including cell differentiation, proliferation, and migration.^19^ In the heart, changes in the expression level of NOX4 were described as enhancing the differentiation of mouse embryonic stem cells into beating cardiomyocytes^29^ although NOX4 knockout mice are born with normal hearts.^17,18^ It should be mentioned that NOX4 can exist as splice variants,^30^ in particular, NOX4D which is expressed in many cell types and localizes to the nucleus, which distinguishes it from NOX4.^31^ Of interest, an observational study of human hearts from ischaemic cardiomyopathy patients indicated a down-regulation of a splice variant consistent with NOX4D.^32^ However, the relevance of NOX4D splice variants to cardiac pathology is still largely unknown.^33^ It was shown in vascular smooth muscle cells that NOX4D increases the phosphorylation of extracellular-signal-regulated kinase1/2 and the nuclear transcription factor Elk-1 and may play a role in nuclear signalling and DNA damage, effects distinct from NOX4.^31^ NOX4D-mediated nuclear ROS production was also reported in cancer cells and COS cells where it may modulate the cell cycle.^34^

NOX2 and NOX4 are both up-regulated during the response to hypertrophic stimuli but appear to have distinct effects in the heart.^35^ NOX2 activation broadly speaking drives a detrimental outcome by amplifying pro-fibrotic, pro-hypertrophic, contractile dysfunction, and cell death pathways, whereas NOX4 is associated with activating a complex protective signalling response (*Figure 1*). Among the features of the adaptive cardiac stress response triggered by NOX4 are the activation of a network of pro-survival transcription factors including HIF1α,^17,36,37^ ATF4,^28^ and nuclear factor erythroid 2-related factor 2 (NRF2),^37–41^ that may all modulate processes of intermediary metabolism—either individually or collectively.^42^

While the major focus of this review is on the effects of NOXs on cardiac intermediary metabolism, it should be noted that NOXs have important roles in other cardiovascular conditions such as hypertension and atherosclerosis that may also impact the heart. The interested reader is directed to comprehensive reviews of these areas.^7,19,43,44^

## 3. Influence of NOXs on the regulation of cardiac glucose and fatty acid oxidation

**Figure 2:**
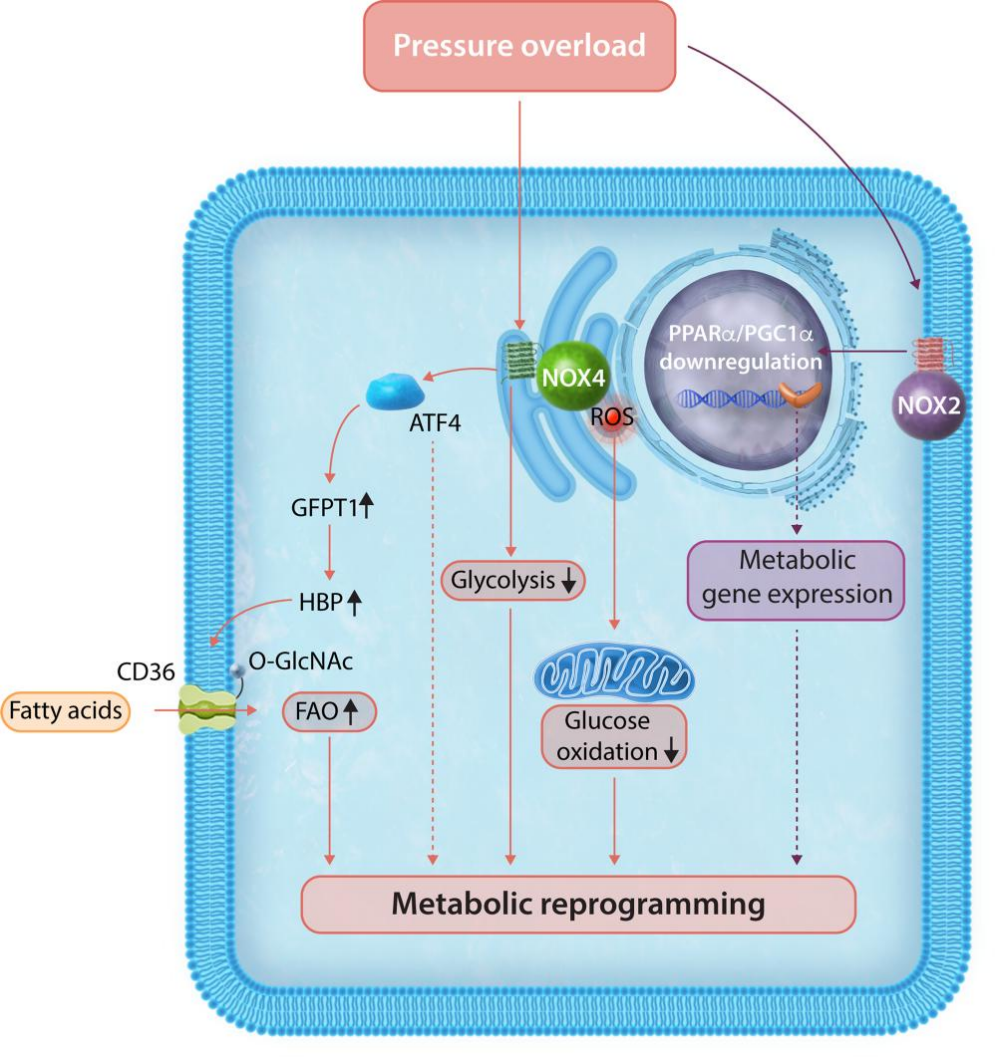
Pressure-overload activates both NOX2 and NOX4 in the heart. NOX2 down-regulates the transcription factor PPARα and PGC1α. This may have subsequent downstream effects on metabolic gene programmes including glucose oxidation and FAO and potential for lipotoxicity. NOX4 augments FAO in cardiomyocytes via an ATF4-dependent mechanism leading to preserved myocardial energetics. Increasing flux through a branch pathway of glycolysis, the HBP, via up-regulation of glutamine-fructose-6-phosphate transaminase 1 (GFPT1) expression leads to the formation of UDP-*N*-acetylglucosamine (UDP-GlcNAc). UDP-GlcNAc is used by *O*-GlcNAc transferase (Ogt) to post-translationally modify specific serine and threonine residues of proteins by O-linked-*N*-acetylglucosaminylation (*O*-GlcNAcylation). Such *O*-GlcNAc modification of Cd36/fatty acid translocase results in an increased uptake and metabolism of fatty acids. It should be noted that broad suppression of NOX-derived ROS causes an up-regulation of PPARα leading to lipotoxicity in the setting of ischaemia–reperfusion injury.

Recently, we identified that NOX4 can drive a substantial rewiring of substrate metabolism in the heart which appears, at least in part, to be transcriptionally mediated and contributes to the adaptive response to chronic haemodynamic overload. We observed that hearts overexpressing NOX4 had reduced glycolysis and glucose oxidation but still had maintained glucose uptake, whereas fatty acid oxidation was significantly increased.^45^ Using targeted metabolomics, we found that there was a build-up of proximal glycolytic intermediates along with evidence of an increased flux through the hexosamine biosynthetic pathway (HBP), resulting in an increase in the production of *O*-GlcNAcylated proteins. This post-translational modification at serine and threonine residues involves the conjugation of a monosaccharide (*N*-acetylglucosamine) derived from the glycolytic intermediate fructose-6-phosphate and may result in specific modifications of protein function or stability.^46,47^ We found that the enhanced HBP activity involves NOX4-mediated activation of ATF4 (*discussed further in a later section*), which increases the level of the rate-limiting HBP enzyme, glutamine fructose-6-phosphate aminotransferase 1 (GFPT1).^45^ Notably, the NOX4-ATF4-dependent increase in protein *O*-GlcNAcylation was linked to the increase in fatty acid oxidation observed in these hearts. We found that the cardiomyocyte fatty acid membrane transporter, CD36, was *O*-GlcNAcylated under these conditions and that this was accompanied by higher fatty acid uptake and oxidation.^45^ Therefore, the shift in cardiac energy substrate utilization from glucose to fatty acids, that is, sustained by NOX4 is related, at least in part, to an ATF4-dependent increase in HBP activity (*Figure 2*).

One of the factors suggested to be involved in the progression from cardiac hypertrophy to heart failure is a decline in fatty acid oxidation that may cause a deficit in ATP production and consequent contractile dysfunction.^48,49^ Augmentation of fatty acid oxidation has been associated with preserving ATP production and ameliorating the effects of pressure-overload-induced failure.^50–52^ Our studies of pressure-overload hypertrophy in NOX4 gene-modified mice support this notion. We found that NOX4-overexpressing hearts not only have a higher fatty acid oxidation both at baseline and after chronic pressure overload but also demonstrate preserved cardiac energetics and have better contractile function and less remodelling than their wild-type counterparts.^53^ A role of NOX4 in augmenting fatty acid oxidation has subsequently also been observed in macrophages,^54^ endothelial cells (ECs),^55^ and hepatocytes,^56^ suggesting that this may be a more general effect of NOX4.

The transcription factor peroxisome proliferator-activated receptor alpha (PPARα) regulates genes involved in fatty acid oxidation and a down-regulation of PPARα is associated with reduced fatty acid oxidation and a transition to heart failure in pressure-overload-induced cardiac hypertrophy. In the study above,^45^ although PPARα levels were not directly assessed, several genes of fatty acid oxidation (FAO) and their protein expression were found to be down-regulated by NOX4 overexpression despite an increase in FAO rates. Therefore, the NOX4-induced changes in FAO appear unrelated to PPARα. However, in a study in which ROS production from both NOX2 and NOX4 was genetically suppressed, an increase in PPARα expression was observed and taken to suggest that basal levels of NOX-derived ROS are required for PPARα regulation.^57^ In this study, overactivity of PPARα was linked to lipotoxicity after ischaemia–reperfusion injury. Another study showed that NOX2 activation in the heart drives PPARα down-regulation which in turn leads to maladaptive pressure-overload hypertrophy.^58^ Indeed, NOX2-deficient hearts did not demonstrate a down-regulation of PPARα, implying that NOX2 is required for down-regulation of PPARα and subsequent cardiac dysfunction. Intriguingly, the authors noted an interesting crosstalk between NOX2 and PPARα, finding that PPARα-deficient hearts failed to show an up-regulation of NOX2 when subjected to pressure-overload hypertrophy, suggesting PPARα regulates NOX2 *in vivo*. The effect of NOX2/PPARα crosstalk on downstream intermediary metabolism was not addressed in this study but does suggest that NOX2 may exert inhibitory effects on fatty acid metabolism through modulation of PPARα.

Peroxisome proliferator-activated receptor gamma coactivator 1-alpha (PGC1α) alongside PPARα is another major regulator of glucose and fatty acid oxidation with its down-regulation being associated with adverse cardiac remodelling.^59^ As described in the next section, NOX2 activation has been associated with the impaired cardiac contractility and mitochondrial dysfunction seen in high-fat diet. A direct assessment of NOX2-dependent changes in substrate handling in hearts has yet to be performed. However, a recent study demonstrated that NOX2 drives activation of Calpain-1 degradation of cardiac Erk5, causing a down-regulation of PGC1α-mediated increases in both glucose and fatty acid oxidation and leading to lipotoxicity.^60^

Interestingly, in skeletal muscle, bouts of moderate exercise can induce cytosolic ROS production from NOX2, resulting in exercise-induced glucose uptake via GLUT4 translocation.^61^ However, it is unknown if NOX2 can regulate cardiac glucose uptake in a similar manner and how this affects downstream intermediary metabolism. In contrast to skeletal muscle NOX2, NOX4 in skeletal muscle ECs was found to be required for metabolic adaptation to chronic exercise, specifically by promoting fatty acid oxidation,^55^ supporting findings that NOX4 regulates FAO in multiple cell types.

An increasingly studied area in heart metabolism is the use of ketone bodies for fuel in addition to the well-described metabolism of glucose and fatty acids. It has been observed in the failing heart that ketone bodies are preferentially oxidized for energy. Indeed, a switch to increasing ketone fuels may ameliorate adverse remodelling in the failing heart and has led to the idea of therapeutic ketosis as a treatment for heart failure.^62,63^ To date, no studies have specifically looked at the role of NOXs in cardiac ketone body metabolism or how ketone bodies affect NOX activity in the heart. Indirectly, global NOX4 deficiency has been associated with ketonaemia, likely to be through its effects on hepatic metabolism,^56^ but the effects on heart biology are unknown.

Taken together, these studies suggest that NOX2 and NOX4 may have opposite effects on FAO in the setting of pressure-overload hypertrophy. Increasing evidence suggests that NOX4 can enhance FAO in a variety of cell types. In contrast, less is known about the role of NOX2 but it seems clear that it can down-regulate cardiac PPARα/PGC1α transcriptional activity and affect subsequent utilization of glucose and fatty acid substrates.

## 4. NOXs in metabolic cardiac disease

NOX2 may indirectly affect metabolism, secondary to increases in oxidative stress and imbalance in redox signalling which leads to mitochondrial dysfunction. Recent studies implicate NOX2 in cardiac dysfunction associated with obesity. A feature of obesity-induced cardiac dysfunction is an increase in myocardial O_2_ consumption and cardiac inefficiency, and it was found that ROS generation from myocardial NOX2 is an important contributor.^64^ The main effect was through increased non-mechanical work and work associated with excitation–contraction coupling. A postulated mechanism may be the induction of mitochondrial uncoupling, leading to O_2_ wasting. The effects of high saturated fat diets and lipotoxicity may in part involve ROS-induced mitochondrial dysfunction, with the consequent development of heart failure. Ablating NOX2 has been shown to reduce high saturated fat and ROS-induced mitochondrial dysfunction in cardiomyocytes.^65^ In addition, another study identified that high-fat diet-induced cardiac hypertrophy was driven by NOX2, with deficiency of NOX2 protecting hearts against LVH.^66^ Another study in an obesogenic model of cardiac dysfunction and fatty acid overload in cardiomyocytes found that NOX2-ROS impaired autophagy by reducing autophagosome clearance, which then led to cardiac dysfunction.^67^ During ischaemia–reperfusion, diabetes and elevated glucose aggravate myocardial injury and cell death. This was found to be improved by inhibiting NOX2 expression. Indeed, NOX2 regulation may be AMPK-dependent as it was shown that increasing AMPK activity could reduce NOX2-dependent oxidative stress and myocardial injury.^68^ Recently hyperglycaemia-associated increases in cardiomyocyte ROS were shown to involve the activation of CaMKII by O-GlcNAcylation and a subsequent generation of ROS by NOX2.^69^

There are some reports that NOX4 may aggravate myocardial injury in models of Type 1 diabetes or in cultured cells treated with high glucose although the mechanism was not fully elucidated.^70^ On the other hand, endothelial NOX4 expression might also protect against adverse cardiac remodelling in experimental diabetes.^71^ Indeed, the diabetic state results in glycated proteins which have been found to induce ROS production from NOX2 rather than NOX4.^72^ Interestingly, in a model of diabetes-induced diastolic dysfunction, both NOX2 and NOX4 expression could be attenuated by AAV6-targeted up-regulation of a constitutively active PI3K, leading to a decrease in ROS and protection against diastolic dysfunction.^73^ However, it was not clear which isoform was pathological in this setting. In another model of diastolic dysfunction induced by diet-induced hypercholesterolaemia, NOX4 was found to be up-regulated relative to NOX2 in the hearts of cholesterol-fed rats.^74^ Furthermore, myocardial microRNA-25 was found to be a direct target for NOX4 and was decreased in cholesterol-fed rat hearts, with *in vitro* studies demonstrating a direct link between reduced microRNA-25, increased NOX4 expression, and oxidative stress. Although microRNAs are also reported to affect NOX2 expression in the heart^75^ and could be exploited to protect against NOX2-driven adverse effects after myocardial infarction,^76^ the effect on metabolic cardiac disease is not known. MicroRNA-488, a known regulator of NOX2 in the heart, is significantly reduced in obesity and would be of interest to investigate with respect to its effects on NOX2 in metabolic cardiac disease.^77^ Overall, the effects of NOX4 are less clear than those of NOX2.

In summary, these studies implicate NOX2 and to a lesser extent NOX4 in metabolic causes of heart failure including obesity, high-fat diet, and diabetes. In general, this appears to involve ROS production induced by the condition (e.g. obesity) and subsequent ROS-induced myocardial injury rather than a direct redox modulation of metabolic pathways.

## 5. NOX, the ISR, and cardiac metabolism

There are numerous published reports that NOX4 is induced during the unfolded protein response (UPR, also known as the ER stress response).^78,79^ This was initially reported in vascular smooth muscle cells during the induction of ER stress and cell death by the oxysterol, 7-ketocholesterol, an oxidized form of LDL, that is, detected at high levels in human atherosclerotic plaques.^80^ Other reports include increased NOX4 expression in response to ER stress agonists such as tunicamycin (an inhibitor of *N*-glycosylation) and thapsigargin (which disrupts ER calcium homeostasis) in cardiac myocytes,^28^ vascular ECs,^12^ and embryonic fibroblasts^28^—suggesting that this may be a universal response across multiple cell types.

The UPR is a highly conserved signalling pathway that senses ER stress—which results in protein misfolding in the organelle—and communicates with the nucleus to promote an adaptive response that rescues folding capacity and restores protein homeostasis.^81–83^ Its importance in the heart is underlined by several studies that show perturbing its homeostatic function leads to cardiovascular disease including heart failure.^84^ The ultimate effects of UPR activation depend upon multiple factors including the duration and intensity of stress, and may result in cell survival, dysfunction, or death. UPR activation comprises three main limbs: the inositol-requiring enzyme 1 alpha—X-box binding protein 1 (IRE1α-XBP1), activating transcription factor 6 (ATF6), and protein kinase R (PKR)-like ER kinase/eukaryotic translation initiation factor 2 alpha (PERK/eIF2α) pathways. IRE1 kinase activation leads to the splicing of XBP1 mRNA and the formation of XBP1s which has potent transcriptional activity. Activation of the transcription factor, ATF6, during the UPR involves its translocation and proteolysis in the Golgi. In the third limb, PERK activation via autophosphorylation leads to the phosphorylation of the α subunit of eIF2α. This, in turn, results in attenuation of global protein synthesis but enhanced translation of a subset of proteins among which the most important is the transcription factor ATF4.^85^ Phosphorylation of eIF2α and increase in ATF4 levels can also be mediated by three other kinases that are activated by diverse stresses unrelated to ER stress, including hypoxia, starvation, viral DNA, and free haem. This overall eIF2α/ATF4 response is termed the integrated stress response (ISR) and drives an extensive pro-survival transcriptional programme that includes a broad range of metabolic pathways such as phospholipid synthesis and increased amino acid biosynthesis and transport.^86^

**Figure 3:**
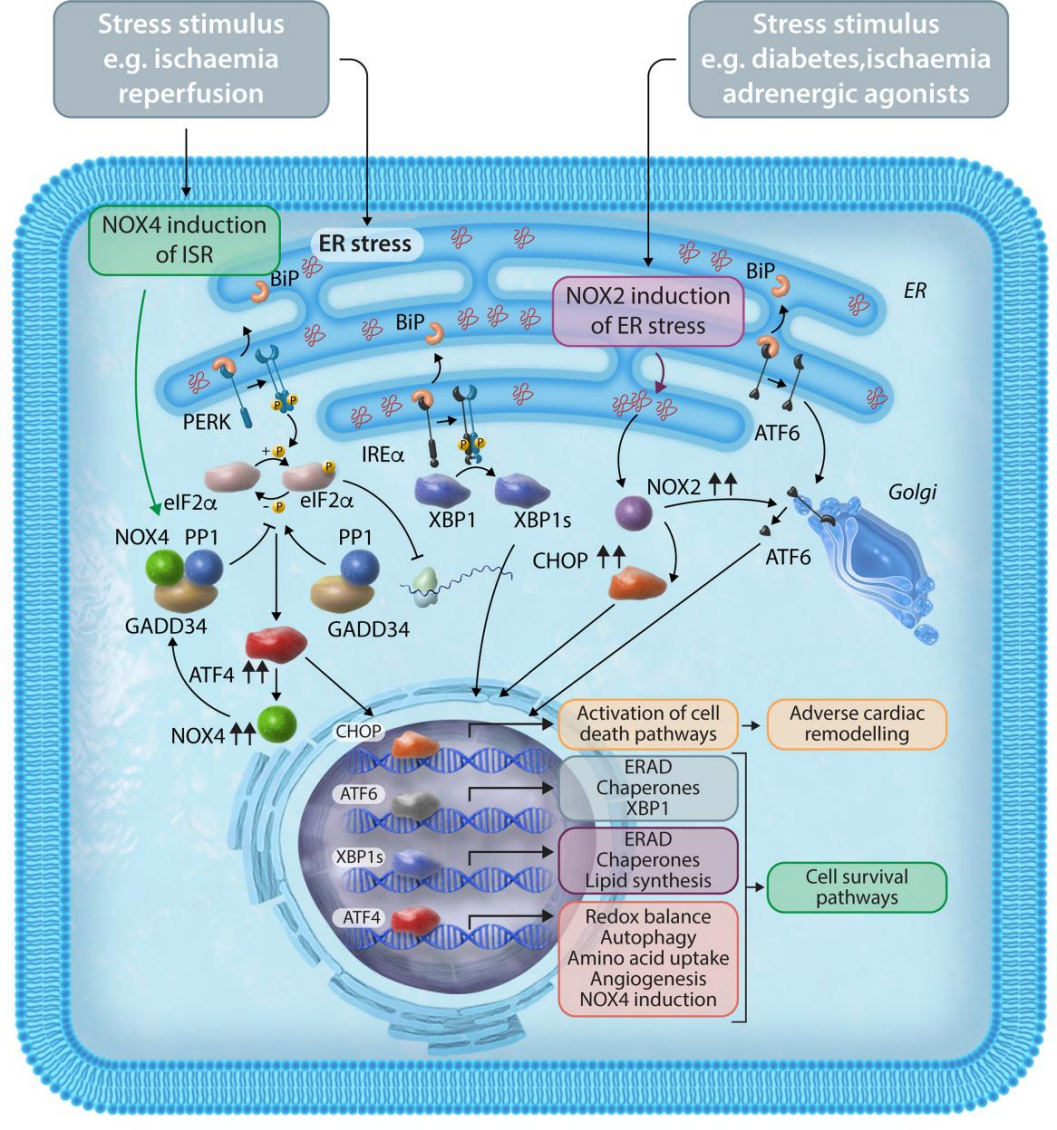
ER stressors activate/induce both NOX2 and NOX4 but lead to different effects on cell fate. ER stress activates the UPR through the interaction of molecular chaperones like BiP with specific pathways that can lead to either cell death or cell survival. NOX2 induces ER stress leading to apoptosis in response to a variety of ER-stress stimuli including diabetes, ischaemia, and adrenergic agonist stimulation. NOX2 can increase levels of CHOP which signals to activation of cell death pathways and adverse cardiac remodelling. NOX2 can also induce ATF6 at least in chronic hyperglycaemic conditions. During ischaemia–reperfusion, ER-located NOX4 can specifically activate the ISR leading to the activation of cell survival pathways. Specifically, NOX4 mediates oxidation of the PP1 metal centre to inactivate the enzyme and sustain eIF2α phosphorylation and ATF4 activation (eIF2α-ATF4 limb). This is independent of the other arms of the UPR, namely the IRE1α-XBP1 pathway (shown for illustration) or the ATF6 pathway. ERAD, ER-associated protein degradation; GADD34, growth arrest and DNA damage-inducible 34; BiP, binding immunoglobulin protein.

Recently, we identified an obligatory role for NOX4 in enhancing eIF2α-ATF4 activation through an intricate localized redox signalling pathway at the ER.^28^ While UPR signalling was evoked by ER stress agonists in cardiac cells (as well as embryonic fibroblasts), the effects of NOX4 were interestingly restricted predominantly to the eIF2α-ATF4 limb of the UPR (*Figure 3*). We found using super-resolution microscopy and cellular fractionation that NOX4 is located predominantly at the ER and that its mRNA levels are increased, in part, via the transcriptional effects of ATF4. The mechanism by which NOX4 augments eIF2α phosphorylation involves the redox inhibition of protein phosphatase-1 (PP1), which normally dephosphorylates eIF2α. We found that NOX4 interacts with the protein, GADD34, which binds to and targets PP1 specifically to the ER. Through the proximity to ER-related PP1 via its binding to growth arrest and DNA damage-inducible 34 (GADD34), NOX4 mediates oxidation of the PP1 metal centre to inactivate the enzyme—a mechanism that contrasts to the cysteine oxidation, that is, the well-established mechanism of redox inhibition of protein tyrosine phosphatases. Notably, this localized inhibition of ER-located PP1 means that other PP1 targets in the cell are unaffected. The consequence of PP1 inhibition at the ER is to sustain eIF2α phosphorylation and ATF4 translation. Thus, NOX4 acts both upstream and downstream of ATF4 to mediate a positive feedback loop to eIF2α-ATF4 signalling.^28^ This NOX4-eIF2α-ATF4 pathway not only enhanced cardiac cell survival in response to ER stress agonists but also mediated cardioprotection and cell survival during heart ischaemia–reperfusion, indicating that NOX4 mediates a broader regulation of the ISR. Overall, this is one of the first detailed mechanisms to be elucidated through which NOX4-dependent compartmentalized redox regulation mediates stress adaptation.^28^

NOX4-dependent regulation of the ISR is likely to have multiple effects on intermediary metabolism. In the study discussed above, among the genes regulated via NOX4-eIF2α-ATF4 signalling were several involved in the transmembrane transport and synthesis of amino acids such as asparagine and glycine.^28^ In ECs, we have shown that an important ATF4 target gene is cystathionine gamma-lyase which acts in the trans-sulfuration pathway that converts cystathionine derived from methionine into cysteine.^87^ The downstream consequences of the modulation of this pathway may include altered cellular glutathione synthesis (which requires cysteine) and therefore redox state, as well as altered production of the signalling molecule hydrogen sulphide which is produced via the action of cystathionine gamma-lyase.^87^ NOX4 was also found to regulate the flux of homocysteine to cysteine in the liver (which will then lead to increased glutathione synthesis) although it is not clear whether this is ATF4 regulated.^88^ In a study investigating cardioprotective effects evoked by aldehyde stress in an *in vivo* mouse model subjected to ischaemia–reperfusion,^89^ it was reported that ATF4 mediated reprogramming of glucose catabolism to increase pentose phosphate pathway (PPP) sugar intermediates and glutathione synthesis—thereby conferring resistance against acute oxidative stress. However, it remains to be established whether NOX4 is involved in this response.

Another important response may be the triggering of autophagy which itself is intricately linked with alterations in cardiac metabolism.^90^ In cardiac myocytes subjected to glucose deprivation, it was shown that NOX4 levels increase in the ER and enhance eIF2α/ATF4 signalling to induce autophagy.^13^ This is in line with prior data that key genes involved in autophagy, such as Unc-51 like autophagy activating kinase 1 (ULK1) and Beclin-1, are transcriptional targets of ATF4 and stimulate autophagy in other cells and tissues such as fibroblasts^91^ and cancer cells.^92^ NOX4-ATF4-dependent enhancement of autophagy appears to be an important protective pathway in the heart as it was found to mediate cardioprotection in response to ischaemia–reperfusion in mice.^13,28^ As mentioned in the previous section, NOX2-ROS can impair autophagy leading to cardiac dysfunction in the setting of obesity^67^—illustrating another instance where the two NOX isoforms have contrasting effects.

In contrast to NOX4, NOX2 may impair myocardial remodelling due to activation of ER stress (*Figure 3*). In the context of diabetes, adverse cardiac remodelling was abrogated by Rac1 deletion in cardiomyocytes leading to less NOX2 activation and PERK/ATF-6 induced ER stress.^93^ In a rabbit model of myocardial infarction, remote myocardial NOX2 expression was found to be increased, in association with increased oxidative stress, elevated C/EBP homologous protein (CHOP), and cleaved ATF6. All the changes were attenuated with apocynin treatment, a non-specific inhibitor of NOX activity, suggesting an association between NOX2, ER stress, and impaired remote myocardial remodelling post-MI.^94^ In cultured cardiomyocytes treated with phenylephrine, either the silencing of NOX2 or its pharmacological inhibition resulted in attenuation of the ER stress response—indicating that phenylephrine-induced ER stress was NOX2 dependent.^95^ Previous studies in macrophages and kidney tissue also demonstrate that ER stress itself can lead to ROS production via NOX2, which in turn can further potentiate CHOP activity leading to apoptosis.^96^ The authors found this was specific for a non-PERK-activated phospho-eIF2α pathway rather than global ER stress. Such a NOX2-driven activation of the UPR/apoptosis pathways may be relevant in the heart, but further studies are required.

In summary, the relationship between NOXs and the ISR is complex. There is an emerging picture for isoform-specific effects, with NOX4-ATF4 pathways driving a cytoprotective ISR through intricate redox signalling and with widespread effects on intermediary metabolism and autophagy. In contrast, NOX2 may enhance ER stress and CHOP-mediated adverse cardiac remodelling.

## 6. NOX enzymes and metabolic regulation during hypoxia

It is well established that HIF1α increases the expression of glycolytic enzymes such as phosphoglycerate kinase 1, glyceraldehyde 3-phosphate dehydrogenase, and lactate dehydrogenase (LDH) during hypoxia^97^ or ischaemia of the heart.^98,99^ HIF1α is also a key regulator of intermediary metabolism in the heart during pathological hypertrophy, directly regulating glycolysis and other aspects of lipid intermediary metabolism.^100^ Numerous studies indicate that NOX4 can regulate HIF activity. Similar to the regulation of ATF4, NOX4 may be both downstream and upstream of HIF. HIF1α transcriptionally activates NOX4 in response to hypoxia in vascular cells,^20^ whereas in renal cell carcinoma NOX4 may help maintain HIF2α expression.^101^ However, NOX4 can also regulate HIF protein levels through the inhibition of prolyl hydroxylase-2 (PHD2, which regulates HIF stability). We found that NOX4-PHD2-dependent up-regulation of cardiomyocyte HIF1α induces vascular endothelial growth factor-mediated adaptive proangiogenic signalling during cardiac hypertrophy.^17^ Similar NOX4-mediated up-regulation of HIF signalling is also reported in microvascular ECs after ischaemia/reperfusion,^102^ in pulmonary artery smooth muscle cells,^103^ and in cerebellar progenitor cells.^104^ In ECs subjected to disturbed flow, it was recently shown that HIF activation downstream of NOX4 drives metabolic reprogramming involving an increase in glycolysis and a decrease in mitochondrial respiratory capacity due to increased expression of pyruvate dehydrogenase kinase-1.^105^ It will be of interest in future studies to also examine the paracrine crosstalk between cardiomyocytes and cardiac ECs during adaptive responses to stress since NOX4-HIF1α signalling occurs in both cell types.^106^

In cardiomyocytes, hypoxia-induced generation was found to be NOX2 mediated, with its inhibition preventing hypoxia-induced apoptosis.^107^ In this study, NOX2 deficiency achieved by adenoviral antisense NOX2 delivery into cardiomyocytes also attenuated hypoxia-induced HIF1α expression. NOX2 can also potentiate HIF signalling in several other cell types. For example, NOX2 activates HIF1α and enhances glycolysis in M1 macrophages.^108^ Part of the mechanism is thought to involve protein tyrosine oxidation and AKT activation causing an increase in glucose uptake. Of note, PHD2 regulation of HIF activity is important in determining macrophage metabolism and function.^109^ In human ECs, NOX2 was found to promote angiogenesis via activation of HIF1α.^110^ In this study, urotensin-II, an angiogenic factor, was found to generate ROS, which could be ablated by inhibiting NOX2 expression. Interestingly, depletion of NOX4 levels unlike NOX2 was less effective in reducing ROS levels in response to urotensin-II. Indeed, vessels from NOX2-deficient mice had a diminished capacity to form new vessels upon urotensin-II stimulation. Interestingly, urotensin-II treatment increased HIF1α expression which when inhibited reduced NOX2 levels, whereas augmenting HIF1α increased NOX2 levels and the associated ROS production. Another interesting finding was that NOX2 itself can regulate HIF1α induced by urotensin-II. The authors conclude that NOX2 is both a HIF1α target gene and is required for increasing HIF1α levels. So as with NOX4, NOX2 can also be upstream and downstream of HIF activation. Studies are required to specifically examine the effect of such activation on intermediary metabolism.

## 7. NOX4 and the activation of NRF2

The transcription factor NRF2 may be activated by oxidative or electrophilic stress as well as several endogenous mediators (e.g. NO, nitro-fatty acids).^111,112^ NRF2 can induce an extensive array of genes involved in cytoprotection, among which are many that are involved in glutathione biosynthesis and several other intermediary metabolic pathways. In other organs, NRF2 regulates intermediary metabolism through glycolytic branch pathways such as the PPP.^113^ In the liver, NRF2 can alter lipogenic as well as gluconeogenic and glycolytic pathways leading to fatty liver disease.^114,115^ In cancer cells, NRF2 activation is linked to increased expression of PPP enzymes and tumour progression.^116,117^

Although NRF2 may in principle be activated by any source of ROS, intriguingly, we have found that endogenous NOX4 is an obligatory activator of NRF2 in the heart both during pathological cardiac hypertrophy^38^ and acute physiological exercise.^39^ As such, NOX4 knockout mice subjected to chronic pressure overload failed to up-regulate the NRF2 pathway and diverse antioxidant and glutathione biosynthetic genes. This was accompanied by evidence of increased oxidative stress and increased mitochondrial DNA damage, indicating that NOX4 regulates redox state in this setting via the up-regulation of NRF2.^38^ The importance of NOX4 in NRF2 activation is not limited to the heart but also evident in mouse embryonic fibroblasts and renal proximal tubular cells.^118^ Intriguingly, there may be crosstalk between NOX2 and NOX4 with down-regulation of NOX4 and NRF2 pathways and up-regulation of NOX2 and NF-kB pathways evident in the hearts and kidneys of animal models with diabetes-induced oxidative stress.^40^ In contrast to these reports, a study in human lung fibroblasts suggested that NOX4 may inhibit NRF2 activation but the mechanism underlying this surprising finding was not defined.^119^

NOX4 is also crucial in maintaining a normal mitochondrial redox state during repeated acute physiological exercise. We found that mice with either a global or cardiac-specific deficiency of NOX4 had a reduced maximal exercise capacity and impaired cardiac contractile function during treadmill running.^39^ The deficiency of NOX4 in the heart led to impaired activation of NRF2, which in turn resulted in an impaired mitochondrial redox state and respiration during acute exercise. A similar phenotype was observed in mice with a cardiac-specific deficiency of NRF2.^39^ Either enhancing NRF2 activation with sulforaphane or augmenting mitochondrial antioxidant capacity by administration of the mitochondrial-targeted antioxidant, Mito-Q, was able to rescue the impaired exercise phenotype in these animals. Therefore, maintaining mitochondrial redox balance via the NOX4-NRF2 axis is a key component of ensuring that the heart can maintain cellular energetics for contractile function during peak exercise. Beyond the maintenance of redox state, whether NOX4 modulates intermediary metabolism or other NRF2-dependent effects in the heart is an interesting question for future work.

Much less has been published regarding the interplay of NOX2 and NRF2. A high fructose diet has been linked to the development of cardiometabolic disease and in a murine model of high fructose diet, myocardial NOX2 in contrast to NOX4 expression was found to be elevated alongside increased hypertrophy and reduced NRF2/KEAP1 expression.^120^ Exercise attenuated NOX2 expression, increased NRF2/KEAP1 ratio, and reduced fructose-mediated hypertrophy.

## 8. NOX4-dependent regulation of mitochondrial calcium homeostasis and cell viability

Recent work indicates an important role for NOX4 in regulating ER to mitochondrial calcium transfer and, through this mechanism, modulating a key cellular pro-survival mechanism.

**Figure 4:**
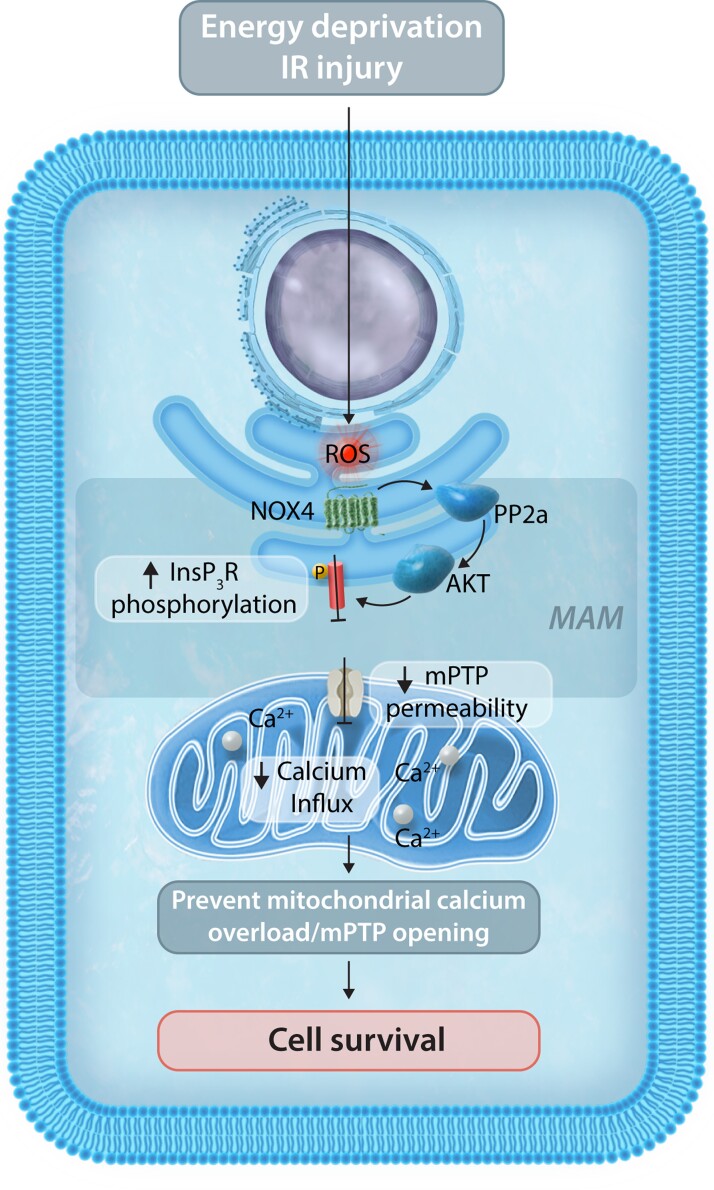
The MAM represents a unique compartment enabling NOX4-localized ROS signalling and ER-mitochondrial calcium communication. Serum starvation or ischaemia–reperfusion (IR) injury can lead to mitochondrial calcium overload and cell death but an increase in NOX4 levels at the MAM prevents excessive calcium transfer between the ER and mitochondria, thereby inhibiting opening of the mitochondrial permeability transition pore and cell death. Mechanistically, NOX4 results in the enhancement of MAM-located Akt activity via a redox inhibition of the phosphatase PP2a. Akt in turn is able to phosphorylate ER InsP3R channels which decrease the release of calcium from the ER to the mitochondria.

We demonstrated the enrichment of NOX4 at the mitochondria-associated membranes (MAM) in several tissues including the heart and human-induced pluripotent stem cell-derived cardiomyocytes.^14^ These sites of contact between the ER and mitochondria are a critical crosstalk platform between the organelles and thus are pivotal in regulating diverse cellular functions, including mitochondrial calcium homeostasis, metabolism, and cell survival.^121^ During severe cell stresses, the MAM may mediate substantial calcium transfer into the mitochondria, resulting in calcium overload and the triggering of mitochondrial permeability transition pore-dependent regulated cell death. The control of this pathway is therefore critical in determining cell survival during many stresses, for example, myocardial ischaemia–reperfusion. We found that NOX4 levels at the MAM increase in response to serum starvation of cardiomyocytes and abrogate calcium transfer from the ER to mitochondria by inhibiting calcium release through the ER inositol trisphosphate receptor channels (InsP_3_R).^14^ This in turn prevents mitochondrial calcium overload and the mPT and enhances cell survival. The mechanism underlying NOX4-mediated inhibition of InsP_3_R involves an enhancement of MAM-located Akt activity via a redox inhibition of the phosphatase PP2a (which normally dephosphorylates Akt). Akt is known to inhibit InsP_3_R via phosphorylation and the increase in Akt activity, therefore, abrogates calcium release (see *Figure 4*). This is another example of NOX4-mediated specific compartmentalized redox signalling. NOX4-mediated inhibition of calcium overload-induced cell death was found to be important in driving protective effects against ischaemia–reperfusion injury in hearts, suggesting a key role for MAM-localized NOX4 signalling in mediating cytoprotective functions. Mitochondrial calcium levels are also a key determinant of mitochondrial metabolism but whether NOX4 modulates this aspect under physiological conditions has not yet been studied.

It should be noted that studies in which NOX4 was overexpressed in cardiac cells have reported that the consequent increase in ROS production may lead to mitochondrial dysfunction and cell death, i.e. effects opposite to those described above.^122^ These apparently discrepant findings may be reconciled by considering that specific compartmentalized signalling (e.g. at the MAM) is dependent upon local low-level ROS production, whereas the overexpression of unphysiologically high levels of NOX4 and ROS will have non-specific damaging effects. Consistent with this idea, it was shown that complete abrogation of NOX-derived ROS production was detrimental during myocardial ischaemia–reperfusion.^57^

The role of NOX2 in mitochondrial calcium homeostasis and cell viability has been studied in sepsis-induced cardiomyopathy.^123^ LPS-induced ROS production requires NOX2 activation. The associated reduction in myocardial contractility could be reversed by NOX2 inhibition which was found to impair sarcoplasmic calcium release. Furthermore, the authors found that *in vivo* LPS-induced myocardial expression of mitochondrial metabolic genes including *Ppara*, *Pgc1a*, and *Cpt1b* was down-regulated, which could be normalized by treatment with apocynin—suggesting NOX2 directly impairs not just calcium handling but also mitochondrial function and metabolism. How NOX2 effects are communicated between ER calcium and mitochondrial function remains unclear in this study.

## 9. NOX and EC metabolism

EC function is intricately linked with cardiac function both by affecting myocardial perfusion and through paracrine crosstalk with cardiomyocytes. EC dysfunction contributes to the pathophysiology of heart failure,^124^ myocardial ischaemia,^125^ and diabetic cardiomyopathy.^126^ NOX enzymes are considered to be the main sources of ROS in ECs. NOX4 is the major isoform expressed in ECs but these cells also express NOX2 and NOX1 (and NOX5 in humans).^127,128^ It is known that ECs rely on glycolysis rather than oxidative metabolism for the majority of their ATP (in contrast to cardiomyocytes).^129^ Indeed, during proliferation, ECs are able to rewire their intermediary metabolism to support growth, including increasing glycolytic flux toward branch pathways such as the PPP and HBP, and harnessing FAO to provide TCA intermediate for the generation of nucleotides.^130^ Both NOX2 and NOX4 have been associated with ROS generation and EC proliferation.^131^ As described in the section on hypoxia, NOX enzymes are also important in regulating EC HIF activity. It is tempting to speculate that NOX activity may be required for the metabolic flexibility of these cells during proliferation, but direct data on this question are lacking. In diabetes, both NOX1 and NOX2 are considered important mediators of vascular dysfunction.^132^ This is in part thought to be due to increased oxidative stress arising from hyperglycaemia induced activation of NOX2. Oxidative stress has subsequent downstream effects on inhibiting glycolysis, increasing proximal metabolites, and feeding into branch pathways and causing defects in EC behaviour.^133^ In contrast to NOX2, NOX4 has a vasoprotective role by maintaining EC function.^134^ This difference is likely to be related to the generation of H_2_O_2_ by NOX4 (whereas NOX2 generates superoxide) and subsequent localized H_2_O_2_-specific signalling which may mediate downstream vasoprotective signalling.^132^ NOX2-derived superoxide reacts with and inactivates NO (also generating peroxynitrite) and may uncouple endothelial eNOS, resulting in amplification of production. In contrast, NOX4-derived H_2_O_2_ does not induce these detrimental effects, especially if H_2_O_2_ levels are not supra-pharmacological.^37^ Moreover, H_2_O_2_ can have direct vasodilator activity, can activate protein kinase G to stimulate vasodilation,^135^ can increase eNOS expression, and can stimulate eNOS activity—all actions that may contribute to vasoprotective activity.^37,136^ Such effects are evident in NOX4-deficient mice where the development of atherosclerosis is enhanced in high-fat diet^137^ and diabetic models.^138^ NOX4 has also been shown to mediate anti-inflammatory properties in the vasculature and the heart, which are most likely underpinned by H_2_O_2_-specific signalling, and contrast with the pro-inflammatory effects of enhanced NOX2 signalling.^137–139^ These effects of NOX4-H_2_O_2_ may be exerted not only at the level of ECs but may also involve the direct modulation of macrophage polarization and phenotype.^140,141^

Given the intricate links between ROS biology and intermediary metabolism, the role of NOX-ROS in regulating intermediary metabolism in ECs is ripe for further exploration. Furthermore, the crosstalk between ECs and cardiomyocytes may be an important mechanism through which cardiac metabolism is also affected, for example, in metabolic cardiac disease.^142,143^

## 10. Summary of NOX effects on cardiac intermediary metabolism

**Figure 5:**
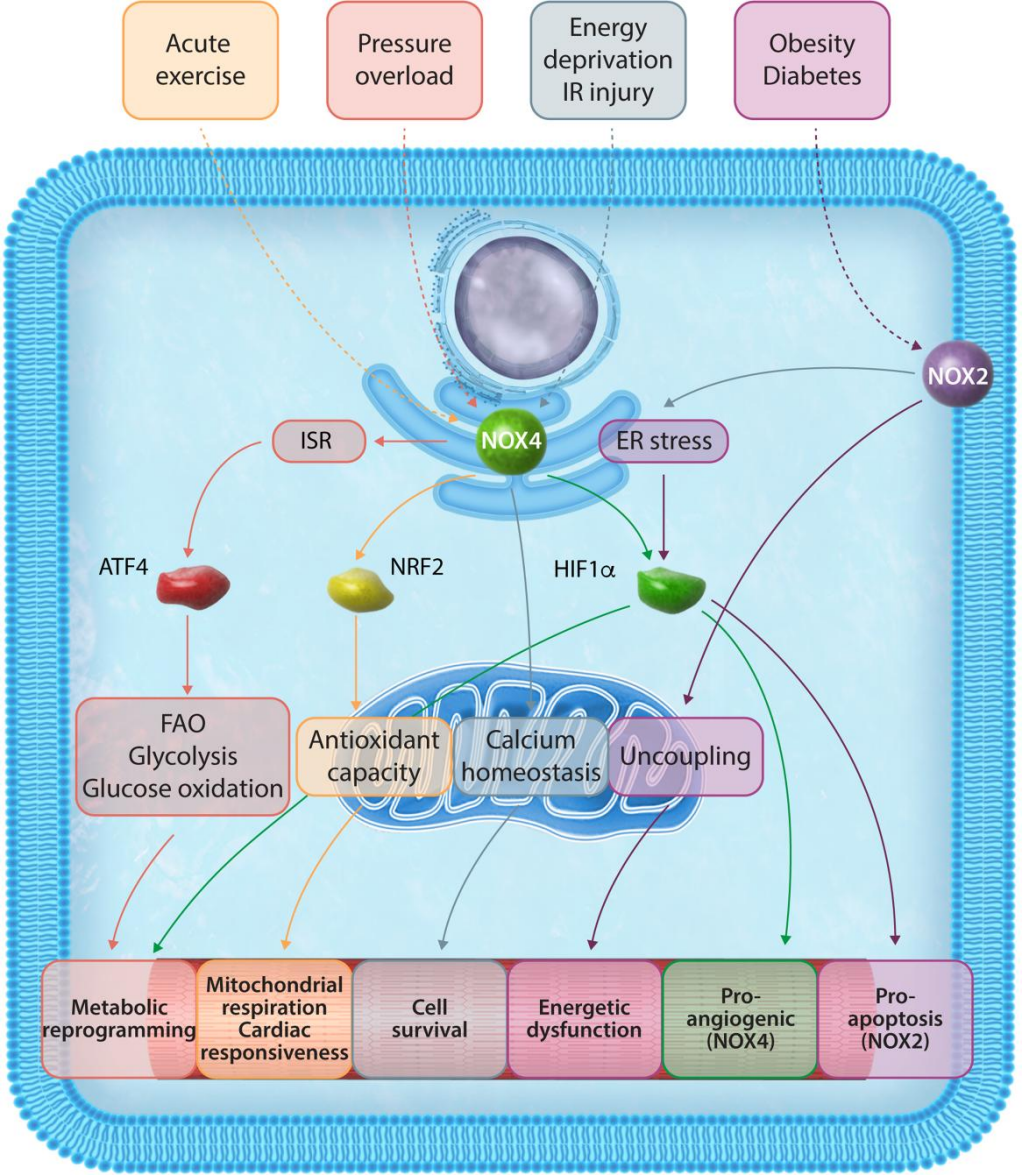
Both NOX2 and NOX4, the main NOX isoforms in cardiomyocytes, can regulate intermediary metabolism in response to a variety of stresses. Several mechanisms are involved, including activation of transcription factors or in the case of NOX4 location at the MAM, targeted ROS signalling to influence mitochondrial function and cell viability. ATF4, activating transcription factor 4; NRF2, nuclear factor erythroid factor 2-related factor 2; HIF1α, hypoxia-inducible factor 1-alpha; ISR, integrated stress response.

The roles of NOX enzymes in redox signalling pathways in the heart are quite well characterized. However, only recently have the effects of NOXs in regulating cardiac metabolism in the heart emerged (*Figure 5*). NOX4 appears to be a crucial component of cellular stress responses and its effects are typically mediated by highly specific and compartmentalized redox signalling dependent both on its subcellular location and the repertoire of proteins that it interacts with under different stress situations. It is notable that such signalling involves the modulation of levels of several transcription factors, including NRF2, ATF4, and HIF1α. Each of these stress-activated transcription factors have direct effects on intermediary metabolism pathways, at least some of which have been found to be regulated by NOX4. In addition, NOX4 may exert metabolic effects through other pathways such as the regulation of ER-to-mitochondrial calcium transfer during severe cell stress. Interestingly, some NOX4 effects are important in physiological settings. For instance, NOX4 is crucial for the hormetic activation of NRF2 in response to acute exercise, with an important impact on mitochondrial redox balance and function and thus exercise capacity. This may comprise a novel physiological mechanism underpinning the benefits of repetitive exercise on mitochondrial ‘fitness’ via NOX4 activation. On the other hand, during chronic stresses such as pressure-overload or ischaemia–reperfusion, NOX4-dependent ATF4 activation drives a broader array of adaptive changes. These include augmentation of fatty acid oxidation with the preservation of energetics, and cytoprotective responses such as the ISR and autophagy. NOX2-mediated effects on metabolism may be especially relevant in obesity, associated with a pro-inflammatory/pro-oxidative response that impairs mitochondrial function. NOX2-ROS generation may be important in driving detrimental ER stress during metabolic cardiac disease. Emerging work also indicates that NOX2 can influence (down-regulate) PPARα and PGC1α, with consequent effects on fatty acid metabolism. However further work is needed to establish how NOX2 regulates these factors, including characterization of the downstream effects on cardiac metabolic pathways.

## 11. Implications and avenues for further research

Intermediary metabolism directly influences several vital functions in tissues. The regulation of NADP/NADPH balance impacts not only on redox state through the regeneration of glutathione but also affects numerous metabolic pathways (e.g. lipid synthesis). Many intermediary metabolic pathways are important in generating cellular building blocks for biosynthesis, such as amino acids, nucleotides, and lipid precursors. They also generate metabolites that may act as signalling molecules.^144^ While the intricacies of these functions have been increasingly established in cancer and other tissues, they remain relatively underexplored in the heart. Alterations in redox state are well established as being an important determinant of chronic changes in cardiac function, for example, during chronic haemodynamic overload or in response to ischaemia–reperfusion. However, less is known about the other aspects of intermediary metabolism. Conceptualization of the heart as a balance between catabolic and anabolic states may provide a useful framework for considering changes such as cardiac remodelling in response to chronic physiological or pathological stress.^1^ It is evident that for the developing or remodelling heart, there will be a need to maintain both growth and contractile function to maintain cardiac output. The regulation of intermediary metabolic pathways may be particularly important in achieving this state.^145^

It is intriguing that NOX enzymes, and NOX4 in particular, appear to impact intermediary metabolic pathways during cardiac stress responses. While the studies to date have begun to outline the upstream components of NOX4-dependent signalling, fully dissecting the links to chronic changes in cardiac structure and function requires further work. NOX4-dependent modulation of protein *O*-GlcNAcylation is especially interesting in relation to intracellular signalling, with prior data in the literature that it may contribute to hypertrophic signalling.^146^ The broader effects of NOX4-dependent ATF4 activation in the heart are also important to elicit, given the evolutionary significance of the ATF4-regulated ISR. Likewise, it will be important to establish how NOX4-mediated regulation of NRF2 fits within the wider scheme of NRF2 regulation and functions in the heart. It is of interest that NOX4 has been associated with driving metabolic alterations in other cell types including cancer cells^16,147^ and macrophages,^54^ suggesting that it could have a fundamental role in modulating cellular intermediary metabolism. Integrative approaches that combine, for example, transcriptomic and proteomic data with metabolic fluxomics are likely to be especially informative in providing a more global picture of the effects in the heart.

It should be recognized that the heart is made up of multiple cell types including cardiomyocytes, fibroblasts, ECs, and immune cells. While we have taken a predominantly cardiomyocyte-centric view in this review, metabolic crosstalk between different cell types may be important in the heart—analogous to emerging data in cancer.^148^ It will be important to develop further research in this area to understand cell-specific effects and the overall impact on cardiac remodelling.

It is probably premature to speculate on the global therapeutic implications of the research findings to date. However, some aspects such as modulation of fatty acid oxidation in the stressed heart or the prevention of mitochondrial calcium overload have obvious relevance to heart failure and ischaemia–reperfusion, respectively. Understanding the role of NOXs in other aspects of substrate metabolism such as ketone body utilization would also be of importance since this is an area of significant therapeutic interest. Given the distinct signalling networks that appear to be activated by NOX2 vs. NOX4, targeting specific isoforms or part of the downstream networks is a potentially attractive proposition to pursue. In this regard, it should be noted that developing specific pharmacological inhibitors of individual NOX isoforms has been challenging to date. Furthermore, the desired aim in many cases might be to enhance rather than inhibit NOX4 signalling. We believe a more comprehensive elucidation of the complex networks regulated by the NOX isoforms and how this affects intermediary metabolism will clarify what are the optimal therapeutic targets to focus on.

## 12. Conclusions

Recent insights into the regulation of key transcription factors (such as NRF2, ATF4, and HIF1α) as well as mitochondrial processes by NOX enzymes suggest that these enzymes have an important role in regulating metabolism, and especially intermediary metabolism, in the heart. The effects of NOX4 are notable in involving highly specific and compartmentalized redox signalling within cells, even extending to physiological settings. These data not only highlight a further facet of NOX biology but also suggest new avenues for research to understand the complexity of chronic changes in cardiac structure and function in an effort to define new therapeutic opportunities for heart disease.

## Data Availability

Data sharing not applicable – no new data generated.

## References

[1] Gibb AA , Hill BG. Metabolic coordination of physiological and pathological cardiac remodeling. Circ Res 2018;123:107–128. 29929976 10.1161/CIRCRESAHA.118.312017 PMC6023588

[2] Doenst T , Nguyen TD, Abel ED. Cardiac metabolism in heart failure: implications beyond ATP production. Circ Res 2013;113:709–724. 23989714 10.1161/CIRCRESAHA.113.300376 PMC3896379

[3] Kolwicz SC , Purohit S, Tian R. Cardiac metabolism and its interactions with contraction, growth, and survival of cardiomyocytes. Circ Res 2013;113:603–616. 23948585 10.1161/CIRCRESAHA.113.302095 PMC3845521

[4] Ritterhoff J , Tian R. Metabolism in cardiomyopathy: every substrate matters. Cardiovasc Res 2017;113:411–421. 28395011 10.1093/cvr/cvx017 PMC5852620

[5] Neubauer S . The failing heart—an engine out of fuel. N Engl J Med 2007;356:1140–1151. 17360992 10.1056/NEJMra063052

[6] Bedard K , Krause KH. The NOX family of ROS-generating NADPH oxidases: physiology and pathophysiology. Physiol Rev 2007;87:245–313. 17237347 10.1152/physrev.00044.2005

[7] Lassègue B , San Martín A, Griendling KK. Biochemistry, physiology, and pathophysiology of NADPH oxidases in the cardiovascular system. Circ Res 2012;110:1364–1390. 22581922 10.1161/CIRCRESAHA.111.243972 PMC3365576

[8] Brandes RP , Weissmann N, Schröder K. Nox family NADPH oxidases: molecular mechanisms of activation. Free Radic Biol Med 2014;76:208–226. 25157786 10.1016/j.freeradbiomed.2014.07.046

[9] Murdoch CE , Zhang M, Cave AC, Shah AM. NADPH oxidase-dependent redox signalling in cardiac hypertrophy, remodelling and failure. Cardiovasc Res 2006;71:208–215. 16631149 10.1016/j.cardiores.2006.03.016

[10] Burgoyne JR , Mongue-Din H, Eaton P, Shah AM. Redox signaling in cardiac physiology and pathology. Circ Res 2012;111:1091–1106. 23023511 10.1161/CIRCRESAHA.111.255216

[11] Prosser BL , Ward CW, Lederer WJ. X-ROS signaling: rapid mechano-chemo transduction in heart. Science 2011;333:1440–1445. 21903813 10.1126/science.1202768

[12] Wu RF , Ma Z, Liu Z, Terada LS. Nox4-derived H_2_O_2_ mediates endoplasmic reticulum signaling through local Ras activation. Mol Cell Biol 2010;30:3553–3568. 20457808 10.1128/MCB.01445-09 PMC2897542

[13] Sciarretta S , Zhai P, Shao D, Zablocki D, Nagarajan N, Terada LS, Volpe M, Sadoshima J. Activation of NADPH oxidase 4 in the endoplasmic reticulum promotes cardiomyocyte autophagy and survival during energy stress through the protein kinase RNA-activated-like endoplasmic reticulum kinase/eukaryotic initiation factor 2α/activating transcription factor 4 pathway. Circ Res 2013;113:1253–1264. 24081881 10.1161/CIRCRESAHA.113.301787 PMC3937770

[14] Beretta M , Santos CX, Molenaar C, Hafstad AD, Miller CC, Revazian A, Betteridge K, Schröder K, Streckfuß-Bömeke K, Doroshow JH, Fleck RA, Su TP, Belousov VV, Parsons M, Shah AM. Nox4 regulates InsP(3) receptor-dependent Ca(2+) release into mitochondria to promote cell survival. EMBO J 2020;39:e103530. 33001475 10.15252/embj.2019103530 PMC7527947

[15] Spencer NY , Yan Z, Boudreau RL, Zhang Y, Luo M, Li Q, Tian X, Shah AM, Davisson RL, Davidson B, Banfi B, Engelhardt JF. Control of hepatic nuclear superoxide production by glucose 6-phosphate dehydrogenase and NADPH oxidase-4. J Biol Chem 2011;286:8977–8987. 21212270 10.1074/jbc.M110.193821 PMC3059029

[16] Shanmugasundaram K , Nayak BK, Friedrichs WE, Kaushik D, Rodriguez R, Block K. NOX4 functions as a mitochondrial energetic sensor coupling cancer metabolic reprogramming to drug resistance. Nat Commun 2017;8:997. 29051480 10.1038/s41467-017-01106-1 PMC5648812

[17] Zhang M , Brewer AC, Schröder K, Santos CX, Grieve DJ, Wang M, Anilkumar N, Yu B, Dong X, Walker SJ, Brandes RP, Shah AM. NADPH oxidase-4 mediates protection against chronic load-induced stress in mouse hearts by enhancing angiogenesis. Proc Natl Acad Sci USA 2010;107:18121–18126. 20921387 10.1073/pnas.1009700107 PMC2964252

[18] Kuroda J , Ago T, Matsushima S, Zhai P, Schneider MD, Sadoshima J. NADPH oxidase 4 (Nox4) is a major source of oxidative stress in the failing heart. Proc Natl Acad Sci USA 2010;107:15565–15570. 20713697 10.1073/pnas.1002178107 PMC2932625

[19] Lassègue B , Griendling KK. NADPH oxidases: functions and pathologies in the vasculature. Arterioscler Thromb Vasc Biol 2010;30:653–661. 19910640 10.1161/ATVBAHA.108.181610 PMC2841695

[20] Diebold I , Petry A, Hess J, Görlach A. The NADPH oxidase subunit NOX4 is a new target gene of the hypoxia-inducible factor-1. Mol Biol Cell 2010;21:2087–2096. 20427574 10.1091/mbc.E09-12-1003 PMC2883952

[21] Zhang L , Sheppard OR, Shah AM, Brewer AC. Positive regulation of the NADPH oxidase NOX4 promoter in vascular smooth muscle cells by E2F. Free Radic Biol Med 2008;45:679–685. 18554521 10.1016/j.freeradbiomed.2008.05.019

[22] Siuda D , Zechner U, El Hajj N, Prawitt D, Langer D, Xia N, Horke S, Pautz A, Kleinert H, Förstermann U, Li H. Transcriptional regulation of Nox4 by histone deacetylases in human endothelial cells. Basic Res Cardiol 2012;107:283. 22791246 10.1007/s00395-012-0283-3

[23] Lyle AN , Deshpande NN, Taniyama Y, Seidel-Rogol B, Pounkova L, Du P, Papaharalambus C, Lassègue B, Griendling KK. Poldip2, a novel regulator of Nox4 and cytoskeletal integrity in vascular smooth muscle cells. Circ Res 2009;105:249–259. 19574552 10.1161/CIRCRESAHA.109.193722 PMC2744198

[24] Prior KK , Wittig I, Leisegang MS, Groenendyk J, Weissmann N, Michalak M, Jansen-Dürr P, Shah AM, Brandes RP. The endoplasmic reticulum chaperone calnexin is a NADPH oxidase NOX4 interacting protein. J Biol Chem 2016;291:7045–7059. 26861875 10.1074/jbc.M115.710772 PMC4807287

[25] Myhill N , Lynes EM, Nanji JA, Blagoveshchenskaya AD, Fei H, Carmine Simmen K, Cooper TJ, Thomas G, Simmen T. The subcellular distribution of calnexin is mediated by PACS-2. Mol Biol Cell 2008;19:2777–2788. 18417615 10.1091/mbc.E07-10-0995 PMC2441662

[26] Takac I , Schröder K, Zhang L, Lardy B, Anilkumar N, Lambeth JD, Shah AM, Morel F, Brandes RP. The E-loop is involved in hydrogen peroxide formation by the NADPH oxidase Nox4. J Biol Chem 2011;286:13304–13313. 21343298 10.1074/jbc.M110.192138 PMC3075677

[27] Nisimoto Y , Diebold BA, Cosentino-Gomes D, Lambeth JD. Nox4: a hydrogen peroxide-generating oxygen sensor. Biochemistry 2014;53:5111–5120. 25062272 10.1021/bi500331y PMC4131900

[28] Santos CX , Hafstad AD, Beretta M, Zhang M, Molenaar C, Kopec J, Fotinou D, Murray TV, Cobb AM, Martin D, Zeh Silva M, Anilkumar N, Schröder K, Shanahan CM, Brewer AC, Brandes RP, Blanc E, Parsons M, Belousov V, Cammack R, Hider RC, Steiner RA, Shah AM. Targeted redox inhibition of protein phosphatase 1 by Nox4 regulates eIF2α-mediated stress signaling. EMBO J 2016;35:319–334. 26742780 10.15252/embj.201592394 PMC4741303

[29] Ateghang B , Wartenberg M, Gassmann M, Sauer H. Regulation of cardiotrophin-1 expression in mouse embryonic stem cells by HIF-1alpha and intracellular reactive oxygen species. J Cell Sci 2006;119:1043–1052. 16507596 10.1242/jcs.02798

[30] Goyal P , Weissmann N, Rose F, Grimminger F, Schäfers HJ, Seeger W, Hänze J. Identification of novel Nox4 splice variants with impact on ROS levels in A549 cells. Biochem Biophys Res Commun 2005;329:32–39. 15721269 10.1016/j.bbrc.2005.01.089

[31] Anilkumar N , San Jose G, Sawyer I, Santos CXC, Sand C, Brewer AC, Warren D, Shah AM. A 28-kDa splice variant of NADPH oxidase-4 is nuclear-localized and involved in redox signaling in vascular cells. Arterioscler Thromb Vasc Biol 2013;33:e104–e112. 23393389 10.1161/ATVBAHA.112.300956

[32] Varga ZV , Pipicz M, Baán JA, Baranyai T, Koncsos G, Leszek P, Kuśmierczyk M, Sánchez-Cabo F, García-Pavía P, Brenner GJ, Giricz Z, Csont T, Mendler L, Lara-Pezzi E, Pacher P, Ferdinandy P. Alternative splicing of NOX4 in the failing human heart. Front Physiol 2017;8:935. 29204124 10.3389/fphys.2017.00935 PMC5698687

[33] Savage N , Anilkumar N, Henckaerts E, Shah AM. The role of cardiomyocyte Nox4D, a redox-active splice variant of NADPH oxidase-4. J Mol Cell Cardiol 2018;120:20.

[34] Scott DA , Schickling BM, Miller FJ. Expression of Nox4 NADPH oxidase splice variants generate hydrogen peroxide and modify the cell cycle. FASEB J 2019;33(S1):815.11.

[35] Zhang M , Perino A, Ghigo A, Hirsch E, Shah AM. NADPH oxidases in heart failure: poachers or gamekeepers? Antioxid Redox Signal 2013;18:1024–1041. 22747566 10.1089/ars.2012.4550 PMC3567780

[36] Zhang M , Mongue-Din H, Martin D, Catibog N, Smyrnias I, Zhang X, Yu B, Wang M, Brandes RP, Schröder K, Shah AM. Both cardiomyocyte and endothelial cell Nox4 mediate protection against hemodynamic overload-induced remodelling. Cardiovasc Res 2018;114:401–408. 29040462 10.1093/cvr/cvx204 PMC6018755

[37] Schröder K , Zhang M, Benkhoff S, Mieth A, Pliquett R, Kosowski J, Kruse C, Luedike P, Michaelis UR, Weissmann N, Dimmeler S, Shah AM, Brandes RP. Nox4 is a protective reactive oxygen species generating vascular NADPH oxidase. Circ Res 2012;110:1217–1225. 22456182 10.1161/CIRCRESAHA.112.267054

[38] Smyrnias I , Zhang X, Zhang M, Murray TVA, Brandes RP, Schröder K, Brewer AC, Shah AM. Nicotinamide adenine dinucleotide phosphate oxidase-4-dependent upregulation of nuclear factor erythroid-derived 2-like 2 protects the heart during chronic pressure overload. Hypertension 2015;65:547–553. 25534702 10.1161/HYPERTENSIONAHA.114.04208

[39] Hancock M , Hafstad AD, Nabeebaccus AA, Catibog N, Logan A, Smyrnias I, Hansen SS, Lanner J, Schröder K, Murphy MP, Shah AM, Zhang M. Myocardial NADPH oxidase-4 regulates the physiological response to acute exercise. eLife 2018;7:e41044. 30589411 10.7554/eLife.41044 PMC6307857

[40] Gajos-Draus A , Duda M, Beręsewicz A. Cardiac and renal upregulation of Nox2 and NF-κB and repression of Nox4 and Nrf2 in season- and diabetes-mediated models of vascular oxidative stress in guinea-pig and rat. Physiol Rep 2017;5:e13474. 29084841 10.14814/phy2.13474 PMC5661235

[41] Brewer AC , Murray TV, Arno M, Zhang M, Anilkumar NP, Mann GE, Shah AM. Nox4 regulates Nrf2 and glutathione redox in cardiomyocytes in *vivo*. Free Radic Biol Med 2011;51:205–215. 21554947 10.1016/j.freeradbiomed.2011.04.022 PMC3112490

[42] Cadenas S . ROS and redox signaling in myocardial ischemia–reperfusion injury and cardioprotection. Free Radic Biol Med 2018;117:76–89. 29373843 10.1016/j.freeradbiomed.2018.01.024

[43] Konior A , Schramm A, Czesnikiewicz-Guzik M, Guzik TJ. NADPH oxidases in vascular pathology. Antioxid Redox Signal 2014;20:2794–2814. 24180474 10.1089/ars.2013.5607 PMC4026218

[44] Takac I , Schröder K, Brandes RP. The Nox family of NADPH oxidases: friend or foe of the vascular system? Curr Hypertens Rep 2012;14:70–78. 22071588 10.1007/s11906-011-0238-3

[45] Nabeebaccus AA , Zoccarato A, Hafstad AD, Santos CXC, Aasum E, Brewer AC, Zhang M, Beretta M, Yin X, West JA, Schröder K, Griffin JL, Eykyn TR, Abel ED, Mayr M, Shah AM. Nox4 reprograms cardiac substrate metabolism via protein O-GlcNAcylation to enhance stress adaptation. JCI Insight 2017;2:e96184. 29263294 10.1172/jci.insight.96184 PMC5752273

[46] Hart GW , Housley MP, Slawson C. Cycling of O-linked beta-N-acetylglucosamine on nucleocytoplasmic proteins. Nature 2007;446:1017–1022. 17460662 10.1038/nature05815

[47] Bond MR , Hanover JA. A little sugar goes a long way: the cell biology of O-GlcNAc. J Cell Biol 2015;208:869–880. 25825515 10.1083/jcb.201501101 PMC4384737

[48] Abdurrachim D , Luiken JJFP, Nicolay K, Glatz JFC, Prompers JJ, Nabben M. Good and bad consequences of altered fatty acid metabolism in heart failure: evidence from mouse models. Cardiovasc Res 2015;106:194–205. 25765936 10.1093/cvr/cvv105

[49] Doenst T , Pytel G, Schrepper A, Amorim P, Färber G, Shingu Y, Mohr FW, Schwarzer M. Decreased rates of substrate oxidation ex *vivo* predict the onset of heart failure and contractile dysfunction in rats with pressure overload. Cardiovasc Res 2010;86:461–470. 20035032 10.1093/cvr/cvp414

[50] Umbarawan Y , Syamsunarno MRAA, Koitabashi N, Obinata H, Yamaguchi A, Hanaoka H, Hishiki T, Hayakawa N, Sano M, Sunaga H, Matsui H, Tsushima Y, Suematsu M, Kurabayashi M, Iso T. Myocardial fatty acid uptake through CD36 is indispensable for sufficient bioenergetic metabolism to prevent progression of pressure overload-induced heart failure. Sci Rep 2018;8:12035. 30104639 10.1038/s41598-018-30616-1 PMC6089997

[51] Smeets PJH , Teunissen BEJ, Willemsen PHM, van Nieuwenhoven FA, Brouns AE, Janssen BJ, Cleutjens JPM, Staels B, van der Vusse GJ, van Bilsen M. Cardiac hypertrophy is enhanced in PPAR alpha−/− mice in response to chronic pressure overload. Cardiovasc Res 2008;78:79–89. 18187461 10.1093/cvr/cvn001

[52] Kolwicz SC Jr , Olson DP, Marney LC, Garcia-Menendez L, Synovec RE, Tian R. Cardiac-specific deletion of acetyl CoA carboxylase 2 prevents metabolic remodeling during pressure-overload hypertrophy. Circ Res 2012;111:728–738. 22730442 10.1161/CIRCRESAHA.112.268128 PMC3434870

[53] Nabeebaccus A , Hafstad A, Eykyn T, Yin X, Brewer A, Zhang M, Mayr M, Shah A. Cardiac-targeted NADPH oxidase 4 in the adaptive cardiac remodelling of the murine heart. Lancet 2015;385(Suppl 1):S73. 26312895 10.1016/S0140-6736(15)60388-9

[54] Moon JS , Nakahira K, Chung KP, DeNicola GM, Koo MJ, Pabón MA, Rooney KT, Yoon JH, Ryter SW, Stout-Delgado H, Choi AMK. NOX4-dependent fatty acid oxidation promotes NLRP3 inflammasome activation in macrophages. Nat Med 2016;22:1002–1012. 27455510 10.1038/nm.4153 PMC5204248

[55] Specht KS , Kant S, Addington AK, McMillan RP, Hulver MW, Learnard H, Campbell M, Donnelly SR, Caliz AD, Pei Y, Reif MM, Bond JM, DeMarco A, Craige B, Keaney JF, Craige SM. Nox4 mediates skeletal muscle metabolic responses to exercise. Mol Metab 2021;45:101160. 33400973 10.1016/j.molmet.2020.101160 PMC7856463

[56] Li Y , Mouche S, Sajic T, Veyrat-Durebex C, Supale R, Pierroz D, Ferrari S, Negro F, Hasler U, Feraille E, Moll S, Meda P, Deffert C, Montet X, Krause KH, Szanto I. Deficiency in the NADPH oxidase 4 predisposes towards diet-induced obesity. Int J Obes 2012;36:1503–1513. 10.1038/ijo.2011.279 22430302

[57] Matsushima S , Kuroda J, Ago T, Zhai P, Ikeda Y, Oka S, Fong GH, Tian R, Sadoshima J. Broad suppression of NADPH oxidase activity exacerbates ischemia/reperfusion injury through inadvertent downregulation of hypoxia-inducible factor-1α and upregulation of peroxisome proliferator-activated receptor-α. Circ Res 2013;112:1135–1149. 23476056 10.1161/CIRCRESAHA.111.300171 PMC3871171

[58] Harvey AP , Robinson E, Edgar KS, McMullan R, O’Neill KM, Alderdice M, Amirkhah R, Dunne PD, McDermott BJ, Grieve DJ. Downregulation of PPARα during experimental left ventricular hypertrophy is critically dependent on Nox2 NADPH oxidase signalling. Int J Mol Sci 2020;21:4406. 32575797 10.3390/ijms21124406 PMC7352162

[59] Montaigne D , Butruille L, Staels B. PPAR control of metabolism and cardiovascular functions. Nat Rev Cardiol 2021;18:809–823. 34127848 10.1038/s41569-021-00569-6

[60] Liu W , Ruiz-Velasco A, Wang S, Khan S, Zi M, Jungmann A, Dolores Camacho-Muñoz M, Guo J, Du G, Xie L, Oceandy D, Nicolaou A, Galli G, Müller OJ, Cartwright EJ, Ji Y, Wang X. Metabolic stress-induced cardiomyopathy is caused by mitochondrial dysfunction due to attenuated Erk5 signaling. Nat Commun 2017;8:494. 28887535 10.1038/s41467-017-00664-8 PMC5591279

[61] Henríquez-Olguin C , Knudsen JR, Raun SH, Li Z, Dalbram E, Treebak JT, Sylow L, Holmdahl R, Richter EA, Jaimovich E, Jensen TE. Cytosolic ROS production by NADPH oxidase 2 regulates muscle glucose uptake during exercise. Nat Commun 2019;10:4623. 31604916 10.1038/s41467-019-12523-9 PMC6789013

[62] Selvaraj S , Kelly DP, Margulies KB. Implications of altered ketone metabolism and therapeutic ketosis in heart failure. Circulation 2020;141:1800–1812. 32479196 10.1161/CIRCULATIONAHA.119.045033 PMC7304522

[63] Takahara S , Soni S, Maayah ZH, Ferdaoussi M, Dyck JRB. Ketone therapy for heart failure: current evidence for clinical use. Cardiovasc Res 2022;118:977–987. 33705533 10.1093/cvr/cvab068

[64] Hafstad AD , Hansen SS, Lund J, Santos CXC, Boardman NT, Shah AM, Aasum E. NADPH oxidase 2 mediates myocardial oxygen wasting in obesity. Antioxidants (Basel) 2020;9:171. 10.3390/antiox9020171 PMC7070669 32093119

[65] Joseph LC , Barca E, Subramanyam P, Komrowski M, Pajvani U, Colecraft HM, Hirano M, Morrow JP. Inhibition of NAPDH oxidase 2 (NOX2) prevents oxidative stress and mitochondrial abnormalities caused by saturated fat in cardiomyocytes. PLoS One 2016;11:e0145750. 26756466 10.1371/journal.pone.0145750 PMC4710525

[66] Bhatti SN , Li JM. Nox2 dependent redox-regulation of Akt and ERK1/2 to promote left ventricular hypertrophy in dietary obesity of mice. Biochem Biophys Res Commun 2020;528:506–513. 32507594 10.1016/j.bbrc.2020.05.162

[67] Jaishy B , Zhang Q, Chung HS, Riehle C, Soto J, Jenkins S, Abel P, Cowart LA, Van Eyk JE, Abel ED. Lipid-induced NOX2 activation inhibits autophagic flux by impairing lysosomal enzyme activity. J Lipid Res 2015;56:546–561. 25529920 10.1194/jlr.M055152 PMC4340303

[68] Wang C , Zhu L, Yuan W, Sun L, Xia Z, Zhang Z, Yao W. Diabetes aggravates myocardial ischaemia reperfusion injury via activating Nox2-related programmed cell death in an AMPK-dependent manner. J Cell Mol Med 2020;24:6670–6679. 32351005 10.1111/jcmm.15318 PMC7299688

[69] Lu S , Liao Z, Lu X, Katschinski DM, Mercola M, Chen J, Heller Brown J, Molkentin JD, Bossuyt J, Bers DM. Hyperglycemia acutely increases cytosolic reactive oxygen species via O-linked GlcNAcylation and CaMKII activation in mouse ventricular myocytes. Circ Res 2020;126:e80–e96. 32134364 10.1161/CIRCRESAHA.119.316288 PMC7210078

[70] Maalouf RM , Eid AA, Gorin YC, Block K, Escobar GP, Bailey S, Abboud HE. Nox4-derived reactive oxygen species mediate cardiomyocyte injury in early type 1 diabetes. Am J Physiol Cell Physiol 2012;302:C597–C604. 22031600 10.1152/ajpcell.00331.2011 PMC3814247

[71] Edgar KS , Gill EK, Patterson E, Hargey CJ, Moez A, Grieve DJ. Adverse cardiac remodelling in experimental diabetes is regulated by endothelial Nox4 NADPH oxidase-derived reactive oxygen species. J Mol Cell Cardiol 2018;120(Suppl):25.

[72] Hansen SS , Aasum E, Hafstad AD. The role of NADPH oxidases in diabetic cardiomyopathy. Biochim Biophys Acta Mol Basis Dis 2018;1864:1908–1913. 28754449 10.1016/j.bbadis.2017.07.025

[73] Prakoso D , De Blasio MJ, Qin C, Rosli S, Kiriazis H, Qian H, Du XJ, Weeks KL, Gregorevic P, McMullen JR, Ritchie RH. Phosphoinositide 3-kinase (p110α) gene delivery limits diabetes-induced cardiac NADPH oxidase and cardiomyopathy in a mouse model with established diastolic dysfunction. Clin Sci 2017;131:1345–1360. 10.1042/CS20170063 28487469

[74] Varga ZV , Kupai K, Szűcs G, Gáspár R, Pálóczi J, Faragó N, Zvara A, Puskás LG, Rázga Z, Tiszlavicz L, Bencsik P, Görbe A, Csonka C, Ferdinandy P, Csont T. MicroRNA-25-dependent up-regulation of NADPH oxidase 4 (NOX4) mediates hypercholesterolemia-induced oxidative/nitrative stress and subsequent dysfunction in the heart. J Mol Cell Cardiol 2013;62:111–121. 23722270 10.1016/j.yjmcc.2013.05.009

[75] Kyrychenko S , Kyrychenko V, Badr MA, Ikeda Y, Sadoshima J, Shirokova N. Pivotal role of miR-448 in the development of ROS-induced cardiomyopathy. Cardiovasc Res 2015;108:324–334. 26503985 10.1093/cvr/cvv238 PMC4648202

[76] Yang J , Brown ME, Zhang H, Martinez M, Zhao Z, Bhutani S, Yin S, Trac D, Xi JJ, Davis ME. High-throughput screening identifies microRNAs that target Nox2 and improve function after acute myocardial infarction. Am J Physiol Heart Circ Physiol 2017;312:H1002–H1012. 28235791 10.1152/ajpheart.00685.2016 PMC5451584

[77] Ait-Aissa K , Nguyen QM, Gabani M, Kassan A, Kumar S, Choi SK, Gonzalez AA, Khataei T, Sahyoun AM, Chen C, Kassan M. MicroRNAs and obesity-induced endothelial dysfunction: key paradigms in molecular therapy. Cardiovasc Diabet 2020;19:136. 10.1186/s12933-020-01107-3 PMC7488343 32907629

[78] Camargo LL , Harvey AP, Rios FJ, Tsiropoulou S, Da Silva RNO, Cao Z, Graham D, McMaster C, Burchmore RJ, Hartley RC, Bulleid N, Montezano AC, Touyz RM. Vascular Nox (NADPH oxidase) compartmentalization, protein hyperoxidation, and endoplasmic reticulum stress response in hypertension. Hypertension 2018;72:235–246. 29844144 10.1161/HYPERTENSIONAHA.118.10824 PMC6004120

[79] Wu RF , Liao C, Hatoum H, Fu G, Ochoa CD, Terada LS. RasGRF couples Nox4-dependent endoplasmic reticulum signaling to Ras. Arterioscler Thromb Vasc Biol 2017;37:98–107. 27856453 10.1161/ATVBAHA.116.307922 PMC5222703

[80] Pedruzzi E , Guichard C, Ollivier V, Driss F, Fay M, Prunet C, Marie JC, Pouzet C, Samadi M, Elbim C, O’Dowd Y, Bens M, Vandewalle A, Gougerot-Pocidalo MA, Lizard G, Ogier-Denis E. NAD(P)H oxidase Nox-4 mediates 7-ketocholesterol-induced endoplasmic reticulum stress and apoptosis in human aortic smooth muscle cells. Mol Cell Biol 2004;24:10703–10717. 15572675 10.1128/MCB.24.24.10703-10717.2004 PMC533993

[81] Santos CXC , Tanaka LY, Wosniak J, Laurindo FR. Mechanisms and implications of reactive oxygen species generation during the unfolded protein response: roles of endoplasmic reticulum oxidoreductases, mitochondrial electron transport, and NADPH oxidase. Antioxid Redox Signal 2009;11:2409–2427. 19388824 10.1089/ars.2009.2625

[82] Ron D , Walter P. Signal integration in the endoplasmic reticulum unfolded protein response. Nat Rev Mol Cell Biol 2007;8:519–529. 17565364 10.1038/nrm2199

[83] Kaufman RJ . Orchestrating the unfolded protein response in health and disease. J Clin Invest 2002;110:1389–1398. 12438434 10.1172/JCI16886 PMC151822

[84] Ren J , Bi Y, Sowers JR, Hetz C, Zhang Y. Endoplasmic reticulum stress and unfolded protein response in cardiovascular diseases. Nat Rev Cardiol 2021;18:499–521. 33619348 10.1038/s41569-021-00511-w

[85] Pavitt GD , Ron D. New insights into translational regulation in the endoplasmic reticulum unfolded protein response. Cold Spring Harb Perspect Biol 2012;4:a012278. 22535228 10.1101/cshperspect.a012278 PMC3367556

[86] Baird TD , Wek RC. Eukaryotic initiation factor 2 phosphorylation and translational control in metabolism. Adv Nutr 2012;3:307–321. 22585904 10.3945/an.112.002113 PMC3649462

[87] Mistry RK , Murray TVA, Prysyazhna O, Martin D, Burgoyne JR, Santos C, Eaton P, Shah AM, Brewer AC. Transcriptional regulation of cystathionine-γ-lyase in endothelial cells by NADPH oxidase 4-dependent signaling. J Biol Chem 2016;291:1774–1788. 26620565 10.1074/jbc.M115.685578 PMC4722457

[88] Murray TVA , Dong X, Sawyer GJ, Caldwell A, Halket J, Sherwood R, Quaglia A, Dew T, Anilkumar N, Burr S, Mistry RK, Martin D, Schröder K, Brandes RP, Hughes RD, Shah AM, Brewer AC. NADPH oxidase 4 regulates homocysteine metabolism and protects against acetaminophen-induced liver damage in mice. Free Radic Biol Med 2015;89:918–930. 26472193 10.1016/j.freeradbiomed.2015.09.015 PMC4698376

[89] Endo J , Sano M, Katayama T, Hishiki T, Shinmura K, Morizane S, Matsuhashi T, Katsumata Y, Zhang Y, Ito H, Nagahata Y, Marchitti S, Nishimaki K, Wolf AM, Nakanishi H, Hattori F, Vasiliou V, Adachi T, Ohsawa I, Taguchi R, Hirabayashi Y, Ohta S, Suematsu M, Ogawa S, Fukuda K. Metabolic remodeling induced by mitochondrial aldehyde stress stimulates tolerance to oxidative stress in the heart. Circ Res 2009;105:1118–1127. 19815821 10.1161/CIRCRESAHA.109.206607

[90] Wang ZV , Hill JA. Protein quality control and metabolism: bidirectional control in the heart. Cell Metab 2015;21:215–226. 25651176 10.1016/j.cmet.2015.01.016 PMC4317573

[91] B’Chir W , Maurin AC, Carraro V, Averous J, Jousse C, Muranishi Y, Parry L, Stepien G, Fafournoux P, Bruhat A. The eIF2α/ATF4 pathway is essential for stress-induced autophagy gene expression. Nucl Acid Res 2013;41:7683–7699. 10.1093/nar/gkt563 PMC3763548 23804767

[92] Pike LR , Singleton DC, Buffa F, Abramczyk O, Phadwal K, Li JL, Simon AK, Murray JT, Harris AL. Transcriptional up-regulation of ULK1 by ATF4 contributes to cancer cell survival. Biochem J 2013;449:389–400. 23078367 10.1042/BJ20120972

[93] Li J , Zhu H, Shen E, Wan L, Arnold JMO, Peng T. Deficiency of rac1 blocks NADPH oxidase activation, inhibits endoplasmic reticulum stress, and reduces myocardial remodeling in a mouse model of type 1 diabetes. Diabetes 2010;59:2033–2042. 20522592 10.2337/db09-1800 PMC2911061

[94] Li B , Tian J, Sun Y, Xu TR, Chi RF, Zhang XL, Hu XL, Zhang YA, Qin FZ, Zhang WF. Activation of NADPH oxidase mediates increased endoplasmic reticulum stress and left ventricular remodeling after myocardial infarction in rabbits. Biochim Biophys Acta 2015;1852:805–815. 25615792 10.1016/j.bbadis.2015.01.010

[95] Zhang N , Wei WY, Yang Z, Che Y, Jin YG, Liao HH, Wang SS, Deng W, Tang QZ. Nobiletin, a polymethoxy flavonoid, protects against cardiac hypertrophy induced by pressure-overload via inhibition of NAPDH oxidases and endoplasmic reticulum stress. Cell Physiol Biochem 2017;42:1313–1325. 28700997 10.1159/000478960

[96] Li G , Scull C, Ozcan L, Tabas I. NADPH oxidase links endoplasmic reticulum stress, oxidative stress, and PKR activation to induce apoptosis. J Cell Biol 2010;191:1113–1125. 21135141 10.1083/jcb.201006121 PMC3002036

[97] Minchenko O , Opentanova I, Caro J. Hypoxic regulation of the 6-phosphofructo-2-kinase/fructose-2,6-bisphosphatase gene family (PFKFB-1-4) expression in *vivo*. FEBS Lett 2003;554:264–270. 14623077 10.1016/s0014-5793(03)01179-7

[98] Moslehi J , Minamishima YA, Shi J, Neuberg D, Charytan DM, Padera RF, Signoretti S, Liao R, Kaelin WG. Loss of hypoxia-inducible factor prolyl hydroxylase activity in cardiomyocytes phenocopies ischemic cardiomyopathy. Circulation 2010;122:1004–1016. 20733101 10.1161/CIRCULATIONAHA.109.922427 PMC2971656

[99] Loor G , Schumacker PT. Role of hypoxia-inducible factor in cell survival during myocardial ischemia–reperfusion. Cell Death Diff 2008;15:686–690. 10.1038/cdd.2008.13 18259200

[100] Krishnan J , Suter M, Windak R, Krebs T, Felley A, Montessuit C, Tokarska-Schlattner M, Aasum E, Bogdanova A, Perriard E, Perriard JC, Larsen T, Pedrazzini T, Krek W. Activation of a HIF1alpha-PPARgamma axis underlies the integration of glycolytic and lipid anabolic pathways in pathologic cardiac hypertrophy. Cell Metab 2009;9:512–524. 19490906 10.1016/j.cmet.2009.05.005

[101] Block K , Gorin Y, Hoover P, Williams P, Chelmicki T, Clark RA, Yoneda T, Abboud HE. NAD(P)H oxidases regulate HIF-2alpha protein expression. J Biol Chem 2007;282:8019–8026. 17200123 10.1074/jbc.M611569200

[102] Wang J , Hong Z, Zeng C, Yu Q, Wang H. NADPH oxidase 4 promotes cardiac microvascular angiogenesis after hypoxia/reoxygenation in vitro. Free Radic Biol Med 2014;69:278–288. 24480752 10.1016/j.freeradbiomed.2014.01.027

[103] Bonello S , Zähringer C, BelAiba RS, Djordjevic T, Hess J, Michiels C, Kietzmann T, Görlach A. Reactive oxygen species activate the HIF-1alpha promoter via a functional NFkappaB site. Arterioscler Thromb Vasc Biol 2007;27:755–761. 17272744 10.1161/01.ATV.0000258979.92828.bc

[104] Eyrich NW , Potts CR, Robinson MH, Maximov V, Kenney AM. Reactive oxygen species signaling promotes hypoxia-inducible factor 1α stabilization in sonic hedgehog-driven cerebellar progenitor cell proliferation. Mol Cell Biol 2019;39:e00268-18. 30692272 10.1128/MCB.00268-18 PMC6447416

[105] Wu D , Huang RT, Hamanaka RB, Krause M, Oh MJ, Kuo CH, Nigdelioglu R, Meliton AY, Witt L, Dai G, Civelek M, Prabhakar NR, Fang Y, Mutlu GM. HIF-1α is required for disturbed flow-induced metabolic reprogramming in human and porcine vascular endothelium. eLife 2017;6:e25217. 28556776 10.7554/eLife.25217 PMC5495571

[106] Zhang M , Shah AM. ROS signalling between endothelial cells and cardiac cells. Cardiovasc Res 2014;102:249–257. 24591150 10.1093/cvr/cvu050

[107] Yu B , Meng F, Yang Y, Liu D, Shi K. NOX2 antisense attenuates hypoxia-induced oxidative stress and apoptosis in cardiomyocyte. Int J Med Sci 2016;13:646–652. 27499697 10.7150/ijms.15177 PMC4974913

[108] Griffiths HR , Gao D, Pararasa C. Redox regulation in metabolic programming and inflammation. Redox Biol 2017;12:50–57. 28212523 10.1016/j.redox.2017.01.023 PMC5312548

[109] Guentsch A , Beneke A, Swain L, Farhat K, Nagarajan S, Wielockx B, Raithatha K, Dudek J, Rehling P, Zieseniss A, Jatho A, Chong M, Santos CXC, Shah AM, Katschinski DM. PHD2 is a regulator for glycolytic reprogramming in macrophages. Mol Cell Biol 2016;37:e00236-16. 27795296 10.1128/MCB.00236-16 PMC5192080

[110] Diebold I , Petry A, Sabrane K, Djordjevic T, Hess J, Görlach A. The HIF1 target gene NOX2 promotes angiogenesis through urotensin-II. J Cell Sci 2012;125:956–964. 22399808 10.1242/jcs.094060

[111] Uruno A , Motohashi H. The Keap1-Nrf2 system as an in *vivo* sensor for electrophiles. Nitric Oxide 2011;25:153–160. 21385624 10.1016/j.niox.2011.02.007

[112] Nguyen T , Nioi P, Pickett CB. The Nrf2-antioxidant response element signaling pathway and its activation by oxidative stress. J Biol Chem 2009;284:13291–13295. 19182219 10.1074/jbc.R900010200 PMC2679427

[113] Hayes JD , Dinkova-Kostova AT. The Nrf2 regulatory network provides an interface between redox and intermediary metabolism. Trends Biochem Sci 2014;39:199–218. 24647116 10.1016/j.tibs.2014.02.002

[114] Slocum SL , Skoko JJ, Wakabayashi N, Aja S, Yamamoto M, Kensler TW, Chartoumpekis DV. Keap1/Nrf2 pathway activation leads to a repressed hepatic gluconeogenic and lipogenic program in mice on a high-fat diet. Arch Biochem Biophys 2016;591:57–65. 26701603 10.1016/j.abb.2015.11.040 PMC4747866

[115] Meakin PJ , Chowdhry S, Sharma RS, Ashford FB, Walsh SV, McCrimmon RJ, Dinkova-Kostova AT, Dillon JF, Hayes JD, Ashford MLJ. Susceptibility of Nrf2-null mice to steatohepatitis and cirrhosis upon consumption of a high-fat diet is associated with oxidative stress, perturbation of the unfolded protein response, and disturbance in the expression of metabolic enzymes but not with insulin resistance. Mol Cell Biol 2014;34:3305–3320. 24958099 10.1128/MCB.00677-14 PMC4135558

[116] Best SA , Ding S, Kersbergen A, Dong X, Song JY, Xie Y, Reljic B, Li K, Vince JE, Rathi V, Wright GM, Ritchie ME, Sutherland KD. Distinct initiating events underpin the immune and metabolic heterogeneity of KRAS-mutant lung adenocarcinoma. Nat Commun 2019;10:4190. 31519898 10.1038/s41467-019-12164-y PMC6744438

[117] Mitsuishi Y , Taguchi K, Kawatani Y, Shibata T, Nukiwa T, Aburatani H, Yamamoto M, Motohashi H. Nrf2 redirects glucose and glutamine into anabolic pathways in metabolic reprogramming. Cancer Cell 2012;22:66–79. 22789539 10.1016/j.ccr.2012.05.016

[118] Nlandu-Khodo S , Dissard R, Hasler U, Schäfer M, Pircher H, Jansen-Durr P, Krause KH, Martin PY, de Seigneux S. NADPH oxidase 4 deficiency increases tubular cell death during acute ischemic reperfusion injury. Sci Rep 2016;6:38598. 27924932 10.1038/srep38598 PMC5141508

[119] Bernard K , Logsdon NJ, Miguel V, Benavides GA, Zhang J, Carter AB, Darley-Usmar VM, Thannickal VJ. NADPH oxidase 4 (Nox4) suppresses mitochondrial biogenesis and bioenergetics in lung fibroblasts via a nuclear factor erythroid-derived 2-like 2 (Nrf2)-dependent pathway. J Biol Chem 2017;292:3029–3038. 28049732 10.1074/jbc.M116.752261 PMC5314196

[120] Alves R , Suehiro CL, Oliveira FG, Frantz EDC, Medeiros RF, Vieira RP, Martins MA, Lin CJ, Nobrega ACL, Toledo-Arruda AC. Aerobic exercise modulates cardiac NAD(P)H oxidase and the NRF2/KEAP1 pathway in a mouse model of chronic fructose consumption. J Appl Physiol 2020;128:59–69. 31647720 10.1152/japplphysiol.00201.2019

[121] Wu S , Zou MH. Mitochondria-associated endoplasmic reticulum membranes in the heart. Arch Biochem Biophys 2019;662:201–212. 30571967 10.1016/j.abb.2018.12.018 PMC6345610

[122] Ago T , Kuroda J, Pain J, Fu C, Li H, Sadoshima J. Upregulation of Nox4 by hypertrophic stimuli promotes apoptosis and mitochondrial dysfunction in cardiac myocytes. Circ Res 2010;106:1253–1264. 20185797 10.1161/CIRCRESAHA.109.213116 PMC2855780

[123] Joseph LC , Kokkinaki D, Valenti MC, Kim GJ, Barca E, Tomar D, Hoffman NE, Subramanyam P, Colecraft HM, Hirano M, Ratner AJ, Madesh M, Drosatos K, Morrow JP. Inhibition of NADPH oxidase 2 (NOX2) prevents sepsis-induced cardiomyopathy by improving calcium handling and mitochondrial function. JCI Insight 2017;2:e94248. 28878116 10.1172/jci.insight.94248 PMC5621873

[124] Paulus WJ , Tschöpe C. A novel paradigm for heart failure with preserved ejection fraction: comorbidities drive myocardial dysfunction and remodeling through coronary microvascular endothelial inflammation. J Am Coll Cardiol 2013;62:263–271. 23684677 10.1016/j.jacc.2013.02.092

[125] Ford TJ , Rocchiccioli P, Good R, McEntegart M, Eteiba H, Watkins S, Shaukat A, Lindsay M, Robertson K, Hood S, Yii E, Sidik N, Harvey A, Montezano AC, Beattie E, Haddow L, Oldroyd KG, Touyz RM, Berry C. Systemic microvascular dysfunction in microvascular and vasospastic angina. Eur Heart J 2018;39:4086–4097. 30165438 10.1093/eurheartj/ehy529 PMC6284165

[126] Tan Y , Zhang Z, Zheng C, Wintergerst KA, Keller BB, Cai L. Mechanisms of diabetic cardiomyopathy and potential therapeutic strategies: preclinical and clinical evidence. Nat Rev Cardiol 2020;17:585–607. 32080423 10.1038/s41569-020-0339-2 PMC7849055

[127] Van Buul JD , Fernandez-Borja M, Anthony EC, Hordijk PL. Expression and localization of NOX2 and NOX4 in primary human endothelial cells. Antioxid Redox Signal 2005;7:308–317. 15706079 10.1089/ars.2005.7.308

[128] Kuroda J , Nakagawa K, Yamasaki T, Nakamura KI, Takeya R, Kuribayashi F, Imajoh-Ohmi S, Igarashi K, Shibata Y, Sueishi K, Sumimoto H. The superoxide-producing NAD(P)H oxidase Nox4 in the nucleus of human vascular endothelial cells. Genes Cells 2005;10:1139–1151. 16324151 10.1111/j.1365-2443.2005.00907.x

[129] De Bock K , Georgiadou M, Carmeliet P. Role of endothelial cell metabolism in vessel sprouting. Cell Metab 2013;18:634–647. 23973331 10.1016/j.cmet.2013.08.001

[130] Schoors S , Bruning U, Missiaen R, Queiroz KCS, Borgers G, Elia I, Zecchin A, Cantelmo AR, Christen S, Goveia J, Heggermont W, Goddé L, Vinckier S, Van Veldhoven PP, Eelen G, Schoonjans L, Gerhardt H, Dewerchin M, Baes M, De Bock K, Ghesquière B, Lunt SY, Fendt SM, Carmeliet P. Fatty acid carbon is essential for dNTP synthesis in endothelial cells. Nature 2015;520:192–197. 25830893 10.1038/nature14362 PMC4413024

[131] Petry A , Djordjevic T, Weitnauer M, Kietzmann T, Hess J, Görlach A. NOX2 and NOX4 mediate proliferative response in endothelial cells. Antioxid Redox Signal 2006;8:1473–1484. 16987004 10.1089/ars.2006.8.1473

[132] Drummond GR , Sobey CG. Endothelial NADPH oxidases: which NOX to target in vascular disease? Trends Endocrinol Metab 2014;25:452–463. 25066192 10.1016/j.tem.2014.06.012

[133] Eelen G , de Zeeuw P, Simons M, Carmeliet P. Endothelial cell metabolism in normal and diseased vasculature. Circ Res 2015;116:1231–1244. 25814684 10.1161/CIRCRESAHA.116.302855 PMC4380230

[134] Eelen G , de Zeeuw P, Treps L, Harjes U, Wong BW, Carmeliet P. Endothelial cell metabolism. Physiol Rev 2018;98:3–58. 29167330 10.1152/physrev.00001.2017 PMC5866357

[135] Burgoyne JR , Madhani M, Cuello F, Charles RL, Brennan JP, Schröder E, Browning DD, Eaton P. Cysteine redox sensor in PKGIa enables oxidant-induced activation. Science 2007;317:1393–1397. 17717153 10.1126/science.1144318

[136] Ray R , Murdoch CE, Wang M, Santos CX, Zhang M, Alom-Ruiz S, Anilkumar N, Ouattara A, Cave AC, Walker SJ, Grieve DJ, Charles RL, Eaton P, Brewer AC, Shah AM. Endothelial Nox4 NADPH oxidase enhances vasodilatation and reduces blood pressure in *vivo*. Arterioscler Thromb Vasc Biol 2011;31:1368–1376. 21415386 10.1161/ATVBAHA.110.219238

[137] Schürmann C , Rezende F, Kruse C, Yasar Y, Löwe O, Fork C, van de Sluis B, Bremer R, Weissmann N, Shah AM, Jo H, Brandes RP, Schröder K. The NADPH oxidase Nox4 has anti-atherosclerotic functions. Eur Heart J 2015;36:3447–3456. 26385958 10.1093/eurheartj/ehv460 PMC4751217

[138] Gray SP , Di Marco E, Kennedy K, Chew P, Okabe J, El-Osta A, Calkin AC, Biessen EAL, Touyz RM, Cooper ME, Schmidt HHHW, Jandeleit-Dahm KAM. Reactive oxygen species can provide atheroprotection via NOX4-dependent inhibition of inflammation and vascular remodeling. Arterioscler Thromb Vasc Biol 2016;36:295–307. 26715682 10.1161/ATVBAHA.115.307012

[139] Murdoch CE , Chaubey S, Zeng L, Yu B, Ivetic A, Walker SJ, Vanhoutte D, Heymans S, Grieve DJ, Cave AC, Brewer AC, Zhang M, Shah AM. Endothelial NADPH oxidase-2 promotes interstitial cardiac fibrosis and diastolic dysfunction through proinflammatory effects and endothelial-mesenchymal transition. J Am Coll Cardiol 2014;63:2734–2741. 24681145 10.1016/j.jacc.2014.02.572

[140] Helfinger V , Palfi K, Weigert A, Schröder K. The NADPH oxidase Nox4 controls macrophage polarization in an NFκB-dependent manner. Oxidat Med Cell Longev 2019;3264858. 10.1155/2019/3264858 PMC6501210 31178956

[141] Mongue-Din H , Patel AS, Looi YH, Grieve DJ, Anilkumar N, Sirker A, Dong X, Brewer AC, Zhang M, Smith A, Shah AM. NADPH oxidase-4 driven cardiac macrophage polarization protects against myocardial infarction-induced remodeling. JACC Basic Transl Sci 2017;2:688–698. 29445778 10.1016/j.jacbts.2017.06.006 PMC5803556

[142] Colliva A , Braga L, Giacca M, Zacchigna S. Endothelial cell-cardiomyocyte crosstalk in heart development and disease. J Physiol 2020;598:2923–2939. 30816576 10.1113/JP276758 PMC7496632

[143] Wan A , Rodrigues B. Endothelial cell-cardiomyocyte crosstalk in diabetic cardiomyopathy. Cardiovasc Res 2016;111:172–183. 27288009 10.1093/cvr/cvw159 PMC4957492

[144] Li F , Xu W, Zhao S. Regulatory roles of metabolites in cell signaling networks. J Genet Genomics 2013;40:367–374. 23876777 10.1016/j.jgg.2013.05.002

[145] Taegtmeyer H , Lubrano G. Rethinking cardiac metabolism: metabolic cycles to refuel and rebuild the failing heart. F1000 Prime Rep 2014;6:90. 10.12703/P6-90 PMC4191265 25374668

[146] Mailleux F , Gélinas R, Beauloye C, Horman S, Bertrand L. O-GlcNAcylation, enemy or ally during cardiac hypertrophy development? Biochim Biophys Acta 2016;1862:2232–2243. 27544701 10.1016/j.bbadis.2016.08.012

[147] Zeng C , Wu Q, Wang J, Yao B, Ma L, Yang Z, Li J, Liu B. NOX4 supports glycolysis and promotes glutamine metabolism in non-small cell lung cancer cells. Free Radic Biol Med 2016;101:236–248. 27989748 10.1016/j.freeradbiomed.2016.10.500

[148] Morandi A , Giannoni E, Chiarugi P. Nutrient exploitation within the tumor-stroma metabolic crosstalk. Trends Cancer 2016;2:736–746. 28741520 10.1016/j.trecan.2016.11.001

